# Information theory for hypergraph similarity

**DOI:** 10.1126/sciadv.aec5619

**Published:** 2026-06-03

**Authors:** Helcio Felippe, Alec Kirkley, Federico Battiston

**Affiliations:** ^1^ Department of Network and Data Science, Central European University, Vienna, Austria.; ^2^ Institute of Data Science, University of Hong Kong, Hong Kong SAR, China.; ^3^ Department of Urban Planning and Design, University of Hong Kong, Hong Kong SAR, China.; ^4^ Urban Systems Institute, University of Hong Kong, Hong Kong SAR, China.; ^5^ Department of AI, Data and Decision Sciences, Luiss University of Rome, Rome, Italy.

## Abstract

Comparing networks is essential for a number of downstream tasks, from clustering to anomaly detection. Despite higher-order interactions being critical for understanding the dynamics of complex systems, traditional approaches for network comparison are limited to pairwise interactions only. Here, we construct a general information theoretic framework for hypergraph similarity, capturing meaningful correspondence among higher-order interactions while correcting for spurious correlations. Our method operationalizes any notion of structural overlap among hypergraphs as a principled normalized mutual information measure, allowing us to derive a hierarchy of increasingly granular formulations of similarity among hypergraphs within and across orders of interactions and at multiple scales. We validate these measures through extensive experiments on synthetic hypergraphs and apply the framework to reveal meaningful patterns in a variety of empirical higher-order networks. Our work provides foundational tools for the principled comparison of higher-order networks, shedding light on the structural organization of networked systems with nondyadic interactions.

## Teaser

Information theoretic principles are used to derive a hierarchy of hypergraph similarity measures.

## INTRODUCTION

Comparing networked systems is central to a variety of downstream tasks in the analysis of complex systems, with applications including clustering, classification, and regression (*1*–*3*). As a result, substantial research has been devoted to developing measures that are capable of capturing similarities in salient structural features of networks (*4*, *5*), with graph similarity measures applied widely across scientific domains spanning biology (*6*), chemistry (*7*), neuroscience (*8*), and sociology (*9*) among others.

Most of the existing network similarity measures are tailored for analyzing graphs consisting solely of pairwise interactions among the entities comprising the nodes in the graph. However, it has been shown through a vast body of recent work that pairwise interactions alone are not sufficient for understanding the structure and dynamics present in many real complex systems, wherein interactions often involve groups of more than two nodes (*10*–*14*). Hypergraphs, which generalize graphs to sets of edges containing any number of nodes (*15*), provide a highly flexible representation for modeling complex systems, allowing for more precise modeling of collective phenomena in contagion and diffusion processes (*16*–*19*), synchronization and evolutionary dynamics (*20*–*22*), and more.

Despite the growing methodological toolkit for analyzing hypergraph data (*23*–*25*), there are relatively few measures for comparing them (*26*–*28*). Many of these methods, including those based on vector embeddings (*29*) and combinations of structural features (*30*), require the specification of free parameters to which the results are highly sensitive, making them challenging to apply in practice without substantial fine-tuning. Meanwhile, methods based on spectral properties (*31*), path lengths (*28*), random walks (*32*), and graphlets (*33*) are capable of capturing complex structural dependencies without tunable parameters but impose a computational complexity that is at least quadratic in the number of nodes in the network, causing them to scale poorly to large systems. In addition, as many of these methods incorporate ad hoc structural features into the similarity calculation with no clear fundamental principles motivating the modeling choices, they provide results that are hard to interpret and may not generalize well to hypergraphs across application domains without substantial modification.

By focusing on the connection between structural regularities in data and its compressibility (*34*), information theory provides a principled foundation on which to build methods for extracting salient structural features in network data in a nonparametric manner. In particular, the minimum description length (MDL) principle, which states that the best model for a dataset is the one that allows for its shortest description in terms of bits of information (*35*), is at the heart of many unsupervised methods for understanding large-scale network structure, including methods for clustering (*36*–*38*), reconstruction and denoising (*39*, *40*), and identifying influential or highly connected groups of nodes (*41*, *42*). By aiming to compress network data based on information encodings that exploit certain structural regularities of interest—e.g., communities, highly connected hub nodes, etc.—MDL-based methods can extract statistically significant structure in graphs while ignoring spurious regularities that arise from statistical noise.

Given its explanatory power and flexibility, information theory has been used in a number of existing works to construct measures for graph comparison (*1*, *43*–*45*), some of which have explicitly used the MDL principle (*46*, *47*). Formulating the problem of graph comparison using the MDL principle allows for fully nonparametric methods that are principled and interpretable. The MDL principle thus provides an ideal framework with which to develop measures of hypergraph similarity.

Here, we introduce a framework for constructing principled and interpretable information-theoretic hypergraph similarity measures using the MDL principle. Within this framework, we derive a series of similarity measures capturing the multiscalar, nested nature of higher-order interactions in an increasingly granular manner. We extend these measures to compute the similarity between a pair of hypergraphs under an arbitrary coarse-graining of the nodes, permitting comparison of interaction patterns across hypergraphs at a desired scale of interest while ignoring fluctuations below the specified scale. Through a range of experiments on real and synthetic hypergraphs, we demonstrate that our framework allows for measures that capture nuanced aspects of structural similarity at multiple scales while remaining robust to statistical noise.

## MEASURES

Analogous to the construction of information theoretic measures for comparing partitions (*48*, *49*), one can construct entropy and conditional entropy measures for any pair of discrete objects by considering different encodings of their structure in an information transmission process. The most natural way to do this is to assign multiple fixed-length codes to transmit the information in the objects at increasing resolution, starting with coarse summary statistics and ending with the final transmission of their detailed structure. The information shared by these objects in their structural overlap can then be quantified using mutual information (MI), which is the amount of information saved in specifying one object when the other is known.

Here, we develop a general framework for hypergraph similarity measures that take the form of normalized MI scores that quantify the amount of shared information between a pair of hypergraphs. By specifying different encoding schemes—that is, different methods for transmitting the network data—we derive a family of MI-based hypergraph similarity measures that capture both intra- and cross-order similarity among hypergraphs. We extend these measures to capture similarity at higher scales using arbitrary coarse-grainings of the nodes.

Given that hypergraphs have higher order interactions than the simple pairwise interactions in normal graphs, there are a number of adaptations one can make to highlight different aspects of similarity within and across different interaction orders. Here, we explore these generalizations in increasing order of complexity to provide a hierarchy of hypergraph MI measures suitable for the comparison of empirical higher-order data.

### MI for graph similarity

Here, we discuss how to construct a simple MI measure for graph comparison (*47*), which will help to motivate the construction of our hypergraph MI measures. For simplicity, we consider input graphs *G*_1_, *G*_2_ that are unweighted and simple (undirected and without self- or multi-edges), although, in principle, it is straightforward to extend the measure to these cases as well by accounting for directed and self-edges by allowing additional valid edge positions or multi-edges with multiset combinatorics [as discussed in (*47*)]. The graphs *G_i_* with i∈{1,2} exist on the same set of *N* labeled nodes, allowing their structural overlap to be unambiguously computed in the computation of the MI, and can be represented as sets of *E*_1_ and *E*_2_ (sorted) tuples respectively in an edgelist representation.

Similar to node partitions, one can construct entropy and conditional entropy measures for the entire graphs *G*_1_, *G*_2_ that consider different encodings of their information for transmission of these objects to a receiver (*48*, *49*). The entropy (or description length) of the graph *G_i_* under a particular encoding scheme is the amount of information it takes to specify *G_i_* after minimizing the codelength over any intermediate representations of *G_i_*—e.g., node communities—for optimal compression. By the Kraft inequality (*50*), for any properly normalized probability distribution *P*(*G*) over some set of valid graphs G, there exists a uniquely decodable prefix code over bitstrings with codelength −log_2_*P*(*G*) for the graph *G*. Therefore, to assign a codelength to *G_i_* to compute its entropy, all we need is a probability distribution over the possible configurations G that *G_i_* may take.

The simplest—and most widely used—encoding is the fixed-length code, which, in this case, assigns all graphs G∈G the same codelength ${|G|}^{-1}$ (*34*). To highlight some structural property of interest about a graph, such as its community structure, one can construct a sequence of fixed-length codes—corresponding to a hierarchical uniform Bayesian prior (*36*)—that use this structural property to achieve compression by reducing the set of possible graphs G in the final encoding step as much as possible. At the same time, one aims to not overcomplicate the encoding to avoid wasting information describing each step. The optimal balance, according to the MDL principle, is achieved when the total codelength (description length) of the observed graph and intermediate encoding steps is minimized.

We can first assume that the receiver knows the total number of nodes *N* and number of edges *E*_1_, *E*_2_ in the two graphs. (These quantities require comparatively negligible information content to specify, so we can safely ignore them.) The simplest encoding is then the fixed-length code over all graphs compatible with these known constraints. There are $\left(\begin{array}{c} \left(\begin{array}{c} N\\ 2 \end{array}\right)\\ E_{i} \end{array}\right)$ possible simple graphs of *E_i_* edges on *N* labeled nodes, and so the entropy (codelength) of graph *G_i_* under this encoding is just

$$H_{\text{graph}}\left(G_{i}\right)=\text{log}\left(\begin{array}{c} \left(\begin{array}{c} N\\ 2 \end{array}\right)\\ E_{i} \end{array}\right)$$ (1)

where we have abbreviated $\text{log}\equiv {\text{log}}_{2}$ for brevity.

In a similar manner to Eq. 1, we can construct a conditional entropy measure between *G*_1_ and *G*_2_, which tells us the amount of information to describe *G*_2_ given that *G*_1_ is known by the receiver (or vice versa). In this case, the Kraft inequality tells us that any probability distribution $P\left(G|G_{1}\right)$ over graphs *G* given the known graph *G*_1_ corresponds to a valid encoding for *G*_2_. However, to achieve compression of *G*_2_ when *G*_1_ is known, we must specify some measure of overlap among the two graphs—without this overlap, our knowledge of *G*_1_ is uninformative and thus not useful for compression. When specifying the overlap, we also have considerable modeling freedom to highlight any structural features of interest. In the simplest case, we can use the set overlap among *G*_1_ and *G*_2_, which counts the number of edges they have in common. Denoting this overlap as

$$E_{12}=\;\left|G_{1}\cap G_{2}\right|$$ (2)

we have that there are $\left(\begin{array}{c} E_{1}\\ E_{12} \end{array}\right)$ possible configurations of the *E*_12_ overlapping edges given that they must be a subset of the *E*_1_ edges in the known *G*_1_. Specifying the overlap $|G_{1}\cap G_{2}|$ therefore costs us $\text{log}\left(\begin{array}{c} E_{1}\\ E_{12} \end{array}\right)$ bits. After receiving this overlap set, the receiver can exclude the *E*_1_ − *E*_12_ edges in *G*_1_ that are not included in the overlap from the possibility of occurring in *G*_2_. Thus, there are $\left(\begin{array}{c} N\\ 2 \end{array}\right)-E_{1}$ remaining edges that could occur in *G*_2_, of which *E*_2_ − *E*_12_ are present, excluding the *E*_12_ we already know from the overlap. Therefore, specifying *G*_2_ given the known overlap $|G_{1}\cap G_{2}|$ requires $\text{log}\left(\begin{array}{c} \left(\begin{array}{c} N\\ 2 \end{array}\right)-E_{1}\\ E_{2}-E_{12} \end{array}\right)$ bits of information. Putting it all together, we have that the conditional entropy of *G*_2_ given *G*_1_ is

$$H_{\text{graph}}\left(G_{2}|G_{1}\right)=\text{log}\left(\begin{array}{c} E_{1}\\ E_{12} \end{array}\right)\left(\begin{array}{c} \left(\begin{array}{c} N\\ 2 \end{array}\right)-E_{1}\\ E_{2}-E_{12} \end{array}\right)$$ (3)

Note that, as with the entropy of Eq. 1, the conditional entropy of Eq. 3 depends on the encoding one chooses—in other words, the way to measure the overlap among *G*_2_ and *G*_1_. We will see in the “Hierarchy of hypergraph similarity measures” section that this allows us to capture similarity among hypergraphs within and across different orders of hyperedges, as well as at different scales of interest.

The difference between Eqs. 1 and 3 quantifies the amount of information we save about *G*_2_ by first knowing *G*_1_ and its overlap with *G*_2_. This is called the MI of *G*_1_ and *G*_2_ (*47*), thus

$${\text{MI}}_{\text{graph}}\left(G_{1};G_{2}\right)=H_{\text{graph}}\left(G_{2}\right)-H_{\text{graph}}\left(G_{2}|G_{1}\right)$$ (4)

and can be used directly as a measure of similarity among the two graphs. When *G*_1_ and *G*_2_ are very similar—i.e., have a high overlap *E*_12_—Eq. 4 will also be high, since knowing *G*_1_ and the overlap will substantially constrain the number of possibilities for *G*_2_ [the conditional entropy $H\left(G_{2}|G_{1}\right)$ will be low]. On the other hand, when *G*_1_ and *G*_2_ are very different [have low overlap *E*_12_ and therefore high $H\left(G_{2}|G_{1}\right)$], the MI will be low because we do not save much information about *G*_2_ by knowing *G*_1_ and the overlap.

Through the Vandermonde identity (*51*), we have

$$\text{log}\left(\begin{array}{c} \sum_{n}y_{n}\\ \sum_{n}x_{n} \end{array}\right)\geq \sum_{n}\text{log}\left(\begin{array}{c} y_{n}\\ x_{n} \end{array}\right)$$ (5)

for any sequences $\left\{x_{n}\right\},\left\{y_{n}\right\}$ of non-negative integers with $y_{n}\geq x_{n}$, which implies that ${\text{MI}}_{\text{graph}}\left(G_{1};G_{2}\right)\geq 0$, such that we will always save information about *G*_2_ by specifying *G*_1_ and the overlap first. [This is a combinatorial version of the concept that conditioning always reduces entropy (*34*).] Equation 4 also has the nice property of being symmetric in the graphs *G*_1_, *G*_2_, as one can show that $H\left(G_{2}\right)-H\left(G_{2}|G_{1}\right)=H\left(G_{1}\right)-H\left(G_{1}|G_{2}\right)$. These two properties—non-negativity and symmetry—are often desirable for MI measures but are not strictly necessary. For example, if one wants to account for the information required to transmit the overlap itself to provide a more accurate accounting of the conditional entropy and reduce finite-size biases, it may sacrifice the symmetry and non-negativity of the MI depending on how the overlap is encoded (*48*, *49*, *52*, *53*). In (*47*), as well as this paper, we ignore the information content of specifying the overlap to ensure non-negativity of the MI measures. However, for the hypergraph case, we will find that breaking the symmetry allows for more encoding flexibility to capture different aspects of overlap.

### Hypergraph normalized MI framework

We consider input hypergraphs *G*_1_, *G*_2_ on the same set of *N* (aligned) nodes that are unweighted and simple, i.e., have no multi- or self-edges. Extensions of our measures to relax the unweighted and simple hypergraph assumptions are conceptually straightforward but involve more complex multiset combinatorics (see section S2). In the hypergraph case, *G*_1_ and *G*_2_ can be represented as edge sets of tuples with two or more nodes, ordered by node index to impose undirectedness. Since hyperedges of different orders have qualitatively different interpretations and impacts on network dynamics (*11*), each hypergraph *G_i_* can be decomposed into “layers” L={2,…,L} such that the layer $G_{i}^{\left(\ell \right)}$ contains all hyperedges of size (order) ℓ in *G_i_*, and *L* is the maximum order of hyperedges across the two hypergraphs *G*_1_, *G*_2_. If no hyperedge of size ℓ exists in *G_i_*, then we set $G_{i}^{\left(\ell \right)}=\left\{\;\right\}$. In section S4, we discuss the case in which the hypergraphs have aligned node labels but different node sets and which preprocessing options are available before computing their similarity.

To account for the nestedness of interactions in our measures, a feature observed in many empirical systems with higher-order interactions (*54*–*57*), we can also let each layer $G_{i}^{\left(\ell^{\prime }\right)}$ be “projected” down onto hyperedges of size $\ell \leq \ell^{\prime }$ by taking the set of all unique subtuples of size ℓ within the tuples of $G_{i}^{\left(\ell^{\prime }\right)}$. We denote the projection from layer $\ell^{\prime }$ to ℓ as $G_{i}^{\left(\ell^{\prime }\to \ell \right)}$, with the convention $G_{i}^{\left(\ell \to \ell \right)}=G_{i}^{\left(\ell \right)}$. For example, if $G_{i}=\left\{(0,1,2),(1,2)\right\}$, then we would have $G_{i}^{\left(3\right)}=\left\{(0,1,2)\right\}$, $G_{i}^{\left(2\right)}=\left\{(1,2)\right\}$, and $G_{i}^{\left(3\to 2\right)}=\left\{(0,1),(1,2),(0,2)\right\}$. We will let the size of (number of hyperedges in) any set $G_{i}^{\left(x\right)}$ be denoted with $E_{i}^{\left(x\right)}=|G_{i}^{\left(x\right)}|$, such that in the previous example we have $E_{i}^{\left(3\right)}=1$, $E_{i}^{\left(2\right)}=1$, $E_{i}^{\left(3\to 2\right)}=3$, and $E_{i}=\sum_{\ell \in L}E_{i}^{\left(\ell \right)}=2$.

We can now define hypergraph MI measures by considering the transmission of the hypergraph *G*_2_ by itself as well as given the known *G*_1_ and some measure(s) of overlap between the two hypergraphs. For generality, we can consider

$$H_{c}\left(G_{i}\right)=\text{log}\left[\#\;\text{possible}\;G_{i}\;\text{under encoding}\;c\right]$$ (6)

and

$$H_{c}\left(G_{j}|G_{i}\right)=\text{log}\left[\#\;\text{possible}\;G_{j}\;\text{under}\;c\;\text{given}\;G_{i}\right]$$ (7)

These expressions reflect the fact that the number of possible configurations of a hypergraph and hence its entropy/conditional entropy depend on what encoding scheme we use—in particular, which constraints are assumed to be known by the receiver under the encoding scheme, and how the encoding scheme defines the overlap among *G*_1_ and *G*_2_. For example, if we let *c* = “graph” be the encoding described in the “MI for graph similarity” section, we recover Eq. 1 from the entropy in Eq. 6 and Eq. 3 from the entropy in Eq. 7. Given Eqs. 6 and 7, we can then construct a MI measure between *G*_1_ and *G*_2_, thus

$${\text{MI}}_{c}\left(G_{1};G_{2}\right)=H_{c}\left(G_{2}\right)-H_{c}\left(G_{2}|G_{1}\right)$$ (8)

To have a uniform scale on which to compare hypergraphs, it is useful to normalize Eq. 8 so that it falls in the range [0, 1], equaling 1 when *G*_1_ and *G*_2_ are identical and a value near *0* when *G*_1_ and *G*_2_ are completely different from each other (i.e., have little overlap). Examining Eq. 8, we can immediately see that $0\leq {\text{MI}}_{c}\left(G_{1};G_{2}\right)\leq H_{c}\left(G_{2}\right)$. The upper bound on the MI results from the fact that the number of configurations of *G*_2_ without any additional constraints from *G*_1_ [$2^{H_{c}\left(G_{2}\right)}$] must be at least as large as the number of configurations of *G*_2_ with additional constraints from *G*_1_ [$2^{H_{c}\left(G_{2}|G_{1}\right)}$]. In addition, the lower bound on the MI results from the non-negativity of the conditional entropy, since its argument (a positive count value) is always at least equal to 1. To allow for full generality in the encodings *c*, we will allow MI*_c_* to potentially be asymmetric, in which case we can construct a symmetric normalized MI measure by taking the maximum of the fractional shared information when considering transmitting *G*_2_ from *G*_1_ and *G*_1_ from *G*_2_. This gives a normalized mutual information (NMI) measure of

$${\text{NMI}}_{c}\left(G_{1},G_{2}\right)=\text{max}\left\{\frac{{\text{MI}}_{c}\left(G_{1};G_{2}\right)}{H_{c}\left(G_{2}\right)},\frac{{\text{MI}}_{c}\left(G_{2};G_{1}\right)}{H_{c}\left(G_{1}\right)}\right\}$$ (9)

$$=1-\text{min}\left\{\frac{H_{c}\left(G_{2}|G_{1}\right)}{H_{c}\left(G_{2}\right)},\frac{H_{c}\left(G_{1}|G_{2}\right)}{H_{c}\left(G_{1}\right)}\right\}$$ (10)

The NMI measure in Eq. 9 is highly flexible, providing a general framework for constructing hypergraph similarity measures.

Equation 9 provides a natural mechanism for assessing the similarity among hypergraphs *G*_1_, *G*_2_ in a manner that is robust to statistical noise. Real-world hypergraphs are typically extremely sparse, only containing a vanishing fraction of the $\left(\begin{array}{c} N\\ \ell \end{array}\right)$ possible hyperedges at each order ℓ, with the sparsity becoming more pronounced as we increase ℓ (*11*). Thus, two hypergraphs *G*_1_, *G*_2_ that are completely uncorrelated—e.g., generated as independent random hypergraphs on *E*_1_ and *E*_2_ hyperedges respectively—will have an overlap that approaches zero for large *N* for any overlap measure that is based on the number of shared tuples among the two hypergraphs or their individual layers (e.g., Eq. 2). We therefore have that *G_i_* and the overlap place very weak constraints on *G_j_*, so that $H_{c}\left(G_{j}\right)\approx H_{c}\left(G_{j}|G_{i}\right)$ and ${\text{NMI}}_{c}\approx 0$. We will more concretely see how this manifests itself in the experiments in Results.

Table 1 summarizes the structure of the general NMI framework we propose for constructing hypergraph similarity measures from fundamental information theoretic principles. While we explore three specific encodings for a natural hierarchy of similarity measures in this paper, our framework applies much more broadly to any meaningful encoding of hypergraph structure.

**Table 1.** Normalized MI framework for constructing hypergraph similarity measures. Descriptions refer to the encodings used in the proposed hierarchy of NMI measures in the “Hierarchy of hypergraph similarity measures” section.

Measure	Description
$H_{c}\left(G_{i}\right)$	Entropy of hypergraph *G_i_*. Amount of information to transmit *G_i_* using an arbitrary lossless encoding *c*.
$H_{c}\left(G_{j}|G_{i}\right)$	Conditional entropy of hypergraph *G_j_* given hypergraph *G_i_*. Amount of information to transmit *G_j_* using lossless encoding *c* when receiver has knowledge of both *G_i_* and a measure of its overlap with *G_j_*.
${\text{MI}}_{c}\left(G_{i};G_{j}\right)$	MI of *G_i_* and *G_j_* (Eq. 8). Amount of information saved when transmitting *G_j_* after knowing *G_i_*, under encoding *c*. Can be asymmetric in general.
${\text{NMI}}_{c}\left(G_{1},G_{2}\right)$	NMI of *G*_1_ and *G*_2_ (Eq. 9). Fraction of information saved when transmitting one hypergraph from another using encoding *c*, under the more efficient order of transmission. Manifestly symmetric hypergraph similarity measure bounded in [0,1].
**Encoding, *c***	**Description**
Bulk	Transmits hypergraphs by specifying hyperedges of all sizes at once. Only accounts for intra-order similarity and is only robust to statistical noise for homogeneous layer densities.
Align	Transmits hypergraphs by specifying hyperedges of each layer separately, with layer $G_{j}^{\left(\ell \right)}$ transmitted using layer $G_{i}^{\left(\ell \right)}$ (and vice versa). Only accounts for intra-order similarity but is robust to statistical noise for any layer densities.
Cross	Transmits hypergraphs by specifying hyperedges of each layer separately, with layer $G_{j}^{\left(\ell \right)}$ transmitted using any layer $G_{i}^{\left(k\right)}$ for k≥ℓ. Accounts for both intra- and cross-order similarity and is robust to statistical noise for any layer densities.

### Hierarchy of hypergraph similarity measures

Perhaps the simplest encoding *c* one can consider is one in which all hyperedges are transmitted at once. We will call this the “bulk” encoding to reflect the one-step transmission. In this case, following the reasoning in the “MI for graph similarity” section, the entropy of each hypergraph is

$$H_{\text{bulk}}\left(G_{i}\right)=\text{log}\left(\begin{array}{c} 2^{N}-N-1\\ E_{i} \end{array}\right)$$ (11)

Here, $2^{N}-N-1$ is the number of possible hyperedges of order at least 2 on *N* nodes, of which we must choose *E_i_* hyperedges to fully specify *G_i_*. In this bulk transmission, the relevant overlap among *G*_1_ and *G*_2_ which will be used for constructing a conditional entropy is just

$$E_{12}=|G_{1}\cap G_{2}|$$ (12)

in which the entire sets *G*_1_ and *G*_2_ are compared at the level of their constituent tuples (analogous to Eq. 2). Then, the conditional entropy under this bulk encoding scheme is given by the logarithm of the number of ways *G_j_* may be configured given its overlap *E*_12_ with *G_i_*, for i,j∈{1,2}. To transmit *G_j_* given knowledge of *G_i_*, we must first specify which subset of *E*_12_ hyperedges among the *E_i_* hyperedges in *G_i_* form the overlap among the two hypergraphs. Then, we must specify the subset of *E_j_* − *E*_12_ remaining hyperedges in *G_j_* that are found among the $\left(2^{N}-N-1\right)-E_{i}$ possible hyperedges outside of *G_i_*. This gives a conditional entropy of

$$H_{\text{bulk}}\left(G_{j}|G_{i}\right)=\text{log}\left(\begin{array}{c} E_{i}\\ E_{12} \end{array}\right)\left(\begin{array}{c} \left(2^{N}-N-1\right)-E_{i}\\ E_{j}-E_{12} \end{array}\right)$$ (13)

for i,j∈{1,2}. The NMI between the hypergraphs *G*_1_ and *G*_2_ under this bulk encoding is then given by substituting Eqs. 11 and 13 into Eq. 9.

Although the bulk encoding provides perhaps the most intuitive way to construct a hypergraph NMI measure using Eq. 9, it has one critical limitation in practice: It considers any subset of the full space of $2^{N}-N-1$ possible hyperedges over *N* nodes to be a valid hypergraph configuration when computing the entropies. Since real hypergraphs often have increasing sparsity as we go to higher and higher layers, the bulk encoding is very inefficient for real hypergraphs *G_i_* since it wastes a substantial amount of space in its codebook assigning bitstrings to hypergraphs that we are unlikely to ever observe. This issue manifests itself, in all but the smallest hypergraphs, with an exaggerated level of similarity between hypergraphs with very little overlap. In this case, since $E_{i}/\left(2^{N}-N-1\right)$ is extremely small, *G*_1_ and *G*_2_ appear as if they share a substantial amount of information for any nonzero overlap *E*_12_ > 0, as it is so unlikely that they have any overlap given the enormous space of all possible hypergraphs considered.

A simple way to correct for this issue is to consider the transmission of each layer of the hypergraphs separately, which, for the conditional entropy, requires an encoding that attributes similarity to the hypergraphs at different layers separately as well. We will use the notation *c* = “align” for the simplest variant of this layer-wise encoding, which computes the similarity among *G*_1_ and *G*_2_ using overlaps between layers of the same order only. There are $\left(\begin{array}{c} N\\ \ell \end{array}\right)$ possible hyperedges in layer ℓ, so if the receiver knows there are $E_{i}^{\left(\ell \right)}$ hyperedges in layer ℓ, we can transmit this layer using $\text{log}\left(\begin{array}{c} \left(\begin{array}{c} N\\ \ell \end{array}\right)\\ E_{i}^{\left(\ell \right)} \end{array}\right)$ bits. To transmit all layers separately, we thus need

$$H_{\text{align}}\left(G_{i}\right)=\sum_{\ell \in L}\text{log}\left(\begin{array}{c} \left(\begin{array}{c} N\\ \ell \end{array}\right)\\ E_{i}^{\left(\ell \right)} \end{array}\right)$$ (14)

bits. An analogous expression can be constructed for the conditional entropy under this encoding by adjusting Eq. 3 for each layer separately, thus

$$H_{\text{align}}\left(G_{j}|G_{i}\right)=\sum_{\ell \in L}\text{log}\left(\begin{array}{c} E_{i}^{\left(\ell \right)}\\ E_{12}^{\left(\ell \right)} \end{array}\right)\left(\begin{array}{c} \left(\begin{array}{c} N\\ \ell \end{array}\right)-E_{i}^{\left(\ell \right)}\\ E_{j}^{\left(\ell \right)}-E_{12}^{\left(\ell \right)} \end{array}\right)$$ (15)

where

$$E_{12}^{\left(\ell \right)}=|G_{1}^{\left(\ell \right)}\cap G_{2}^{\left(\ell \right)}|$$ (16)

is the overlap of the ℓth layer in *G*_1_ and the ℓth layer in *G*_2_. Subbing into Eq. 9 then gives an NMI measure ${\text{NMI}}_{\text{align}}\left(G_{1},G_{2}\right)$ between hypergraphs *G*_1_ and *G*_2_ under this refined encoding. By assessing the similarity at each layer separately to construct the conditional entropy, this encoding also naturally accounts for sparsity differences across the layers and provides a more granular description of similarity among two hypergraphs than the bulk encoding.

Both the bulk and align encodings are capable of capturing similarity between hypergraphs occurring within the same order of hyperedges (i.e., intra-order similarity) but fail to capture similarity between hypergraphs that can occur across different orders of hyperedges (i.e., cross-order similarity). For instance, dyadic interactions in hypergraph *G*_1_ might be similar to (subsets of) larger groups interactions in *G*_2_, but the two previous measures would assign to *G*_1_ and *G*_2_ a low similarity score. It is therefore useful to refine the encoding formulation to construct a more flexible measure, able to capture higher-order similarity not only within but also across multiple orders of interaction simultaneously.

The unnormalized MI measures ${\text{MI}}_{\text{bulk}}\left(G_{1};G_{2}\right)$ and ${\text{MI}}_{\text{align}}\left(G_{1};G_{2}\right)$ (Eq. 8) will be symmetric in the input hypergraphs *G*_1_, *G*_2_. Thus, for these cases, the normalization of Eq. 9 is equivalent to normalizing the MI by the smaller of the two hypergraph entropies $\text{min}\left(H_{c}\left(G_{1}\right),H_{c}\left(G_{2}\right)\right)$. While this symmetry is often desirable for an NMI measure of similarity, allowing the MI to be asymmetric by using the more general form of the NMI in Eq. 9 allows for greater flexibility in the encodings one uses. Specifically, it allows for the transmission of information across nested hyperedges of different orders, since the information to transmit lower-order hyperedges from higher-order hyperedges is different (typically much lower) than the information to transmit higher-order hyperedges from lower-order hyperedges—there are many higher-order hyperedges compatible with (i.e., that are a superset of) a given set of lower-order hyperedges, and higher-order interactions cannot be uniquely determined from lower-order interactions alone (*58*). Meanwhile, there are comparatively few lower-order hyperedges that are a subset of a given higher-order hyperedge, reducing the information cost to go “downward” from higher- to lower-order relative to “upward” (the opposite direction). This natural asymmetry that arises in cross-layer encodings is accommodated by the normalization of Eq. 9, as it takes the better of the two directions when quantifying similarity.

Beyond this inherent information asymmetry that typically favors downward transmission in a cross-layer encoding, it is also computationally much more efficient to consider only the downward direction of transmission from a higher-order layer *k* to a lower-order layer ℓ and not vice versa. There are fast ways to exactly compute the cardinality of a projection $G_{i}^{\left(k\to \ell \right)}$ as well as its overlap with a lower order layer $G_{j}^{\left(\ell \right)}$ of another hypergraph, see section S1 for details. Meanwhile, there is likely no fast algorithm for the reverse direction of transmission unless one sacrifices a substantial amount of data compression. This is because an efficient encoding will require using multiple hyperedges in $G_{j}^{\left(\ell \right)}$ to transmit each hyperedge in $G_{i}^{\left(k\right)}$—otherwise, there are at least $\left(\begin{array}{c} N-\ell \\ k-\ell \end{array}\right)$ remaining choices for the nodes in each hyperedge of $G_{i}^{\left(k\right)}$, since only ℓ nodes can be accounted for by a single edge in $G_{j}^{\left(\ell \right)}$. The resulting encoding would have a non-negligible information cost to map each higher-order hyperedge in $G_{i}^{\left(k\right)}$ to a set of lower-order hyperedges in $G_{j}^{\left(\ell \right)}$ and would also require a matching algorithm for optimizing the encoding cost, sacrificing the computational efficiency of the method.

Given the previous considerations of information asymmetry and computational complexity, we develop a cross-layer encoding that permits the transmission of layer $G_{j}^{\left(\ell \right)}$ from any layer $G_{i}^{\left(k\right)}$, so long as ℓ≤k. To allow for a layer $G_{i}^{\left(k\right)}$ in *G_i_* to aid in the transmission of a layer $G_{j}^{\left(\ell \right)}$ of a different order in *G_j_*, we consider the overlap of the projected layer $G_{i}^{\left(k\to \ell \right)}$ and $G_{j}^{\left(\ell \right)}$. We can therefore define an overlap measure for conditional entropies that incorporates cross-layer similarity as

$$E_{i\to j}^{\left(k\to \ell \right)}=|G_{i}^{\left(k\to \ell \right)}\cap G_{j}^{\left(\ell \right)}|,k\geq \ell$$ (17)

Modifying Eq. 3 appropriately then gives the layer-wise conditional entropy

$$\text{log}\left(\begin{array}{c} E_{i}^{\left(k\to \ell \right)}\\ E_{i\to j}^{\left(k\to \ell \right)} \end{array}\right)\left(\begin{array}{c} \left(\begin{array}{c} N\\ \ell \end{array}\right)-E_{i}^{\left(k\to \ell \right)}\\ E_{j}^{\left(\ell \right)}-E_{i\to j}^{\left(k\to \ell \right)} \end{array}\right)$$ (18)

Now, if we aim to transmit *G_j_* in the most efficient way possible under this encoding structure, we should transmit $G_{j}^{\left(\ell \right)}$ from the layer $k_{i}^{\left(\ell \right)}$ in *G_i_* under which this layer-wise conditional entropy is minimized. More formally, the best layer $k_{i}^{\left(\ell \right)}$ is given by

$$k_{i}^{\left(\ell \right)}=\underset{k\geq \ell }{\text{arg}\;\text{min}}\left\{\text{log}\left(\begin{array}{c} E_{i}^{\left(k\to \ell \right)}\\ E_{i\to j}^{\left(k\to \ell \right)} \end{array}\right)\left(\begin{array}{c} \left(\begin{array}{c} N\\ \ell \end{array}\right)-E_{i}^{\left(k\to \ell \right)}\\ E_{j}^{\left(\ell \right)}-E_{i\to j}^{\left(k\to \ell \right)} \end{array}\right)\right\}$$ (19)

Putting it all together, we can construct a normalized MI NMI_cross_ that incorporates cross-layer similarity as follows. For the entropy, we can use the same expression as in Eq. 14, giving

$$H_{\text{cross}}\left(G_{i}\right)=\sum_{\ell \in L}\text{log}\left(\begin{array}{c} \left(\begin{array}{c} N\\ \ell \end{array}\right)\\ E_{i}^{\left(\ell \right)} \end{array}\right)$$ (20)

Also, for the conditional entropy under this encoding, we can modify Eq. 15 appropriately to account for the best layer $k_{i}^{\left(\ell \right)}$ in *G_i_* with which to transmit layer $G_{j}^{\left(\ell \right)}$. The resulting expression is

$$H_{\text{cross}}\left(G_{j}|G_{i}\right)=\sum_{\ell \in L}\text{log}\left(\begin{array}{c} E_{i}^{\left(k_{i}^{\left(\ell \right)}\to \ell \right)}\\ E_{i\to j}^{\left(k_{i}^{\left(\ell \right)}\to \ell \right)} \end{array}\right)\left(\begin{array}{c} \left(\begin{array}{c} N\\ \ell \end{array}\right)-E_{i}^{\left(k_{i}^{\left(\ell \right)}\to \ell \right)}\\ E_{j}^{\left(\ell \right)}-E_{i\to j}^{\left(k_{i}^{\left(\ell \right)}\to \ell \right)} \end{array}\right)$$ (21)

As before, subbing the entropy and conditional entropy into Eq. 9 gives the NMI under this cross-layer encoding. The NMI_cross_ measure is the most flexible and nuanced of the measures we present here. Hence, it is the primary NMI measure of focus for the experiments in Results.

It is worth noting that any NMI measure constructed using the framework in the “Hypergraph normalized MI framework” section—and thus the three measures NMI_bulk_, NMI_align_, and NMI_cross_ of this section—will give a maximum score of 1 for isomorphic hypergraphs *G*_1_, *G*_2_ when their node labels are correctly aligned. This is because, once the (complete) structural overlap among *G*_1_, *G*_2_ is known, we know everything about *G*_2_ after knowing *G*_1_ and vice versa. However, nonisomorphic hypergraphs can also obtain the maximum similarity score of 1 for NMI_cross_ (but not NMI_bulk_ or NMI_align_), since similarity is assessed across layers. Specifically, ${\text{NMI}}_{\text{cross}}\left(G_{1},G_{2}\right)=1$ for any pair of hypergraphs *G*_1_, *G*_2_ for which each of the layers in one hypergraph are fully nested within at least one layer of the other hypergraph. This flexibility is crucial for assessing similarity beyond pure structural isomorphism, allowing for the nested structures ubiquitous in real hypergraphs to contribute to their similarity. Moreover, such nestedness is critical for understanding structural redundancy in higher-order systems (*59*–*61*). In this context, information encodings that account for nested structures can be used to understand which layers of a hypergraph are most critical for summarizing its higher-order structure (*62*, *63*).

**Fig. 1.**
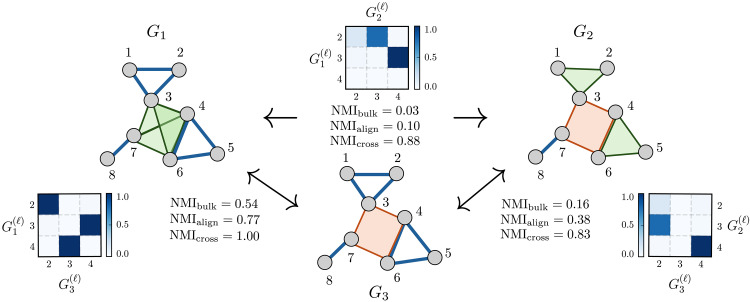
Hierarchy of information-theoretic measures for hypergraph similarity. Hypergraphs *G*_1_, *G*_2_, and *G*_3_ are defined on the same set of *N* = 8 labeled nodes, with hypergraph layers $G_{i}^{\left(\ell \right)}$ indexed by ℓ∈{2,3,4} and illustrated as thick blue lines (dyads), green triangles (triplets), and orange squares (quadruplets). Heatmaps show the order-order MI between pairwise projections of hypergraph layers $G_{i}^{\left(\ell \right)}$ and $G_{j}^{\left(k\right)}$, for all ℓ,k∈{2,3,4}. The three proposed hypergraph MI measures—NMI_bulk_, NMI_align_, NMI_cross_, which are derived using the general framework discussed in the “Hierarchy of hypergraph similarity measures” section—are shown for each pair of hypergraphs. These measures assess the structural similarity between a pair of hypergraphs with increasingly detailed encodings to highlight structural overlaps at and across different hyperedge orders.

In Fig. 1, we show the results of applying our measures to three small example hypergraphs on the same set of *N* = 8 nodes. We can see that all three hypergraphs generally share similar structure across their layers but that the three measures vary substantially across all pairs. Next to each hypergraph pair, for reference, we plot matrices showing the graph NMI measure of (*47*) applied to the projections of each layer to the lower order of the two. These order-order similarity matrices are defined with entries

$$I_{\ell \ell^{\prime }}=h_{b}\left(p_{\ell }\right)+h_{b}\left(p_{\ell^{\prime }}\right)-h_{s}\left(P_{\ell \ell^{\prime }}\right)$$ (22)

where the orders satisfy $\ell^{\prime }\geq \ell$, $h_{b}\left(x\right)=-p\text{log}p-\left(1-p\right)\text{log}\left(1-p\right)$ is the binary entropy, $h_{s}\left(x\right)=-\sum_{i}x_{i}\text{log}x_{i}$ is the Shannon entropy, and $P_{\ell \ell^{\prime }}=\left\{p_{\ell \ell^{\prime }},p_{\ell }-p_{\ell \ell^{\prime }},p_{\ell^{\prime }}-p_{\ell \ell^{\prime }},1-p_{\ell }-p_{\ell^{\prime }}+p_{\ell \ell^{\prime }}\right\}$ is a vector totaling the overlaps among the layers (analogous to a confusion matrix), with $p_{\ell }=|G^{\left(\ell \right)}|/\left(\begin{array}{c} N\\ \ell \end{array}\right)$, $p_{\ell^{\prime }}=|G^{\left(\ell^{\prime }\to \ell \right)}|/\left(\begin{array}{c} N\\ \ell \end{array}\right)$, and $p_{\ell \ell^{\prime }}=|G^{\left(\ell^{\prime }\to \ell \right)}\cap G^{\left(\ell \right)}|/\left(\begin{array}{c} N\\ \ell \end{array}\right)$ the relevant densities of hyperedges used to compute the entries.

The hypergraphs *G*_1_ and *G*_2_ are similar in that $G_{1}^{\left(2\right)}$ has a high structural overlap with $G_{2}^{\left(3\right)}$, and $G_{1}^{\left(3\right)}$ (with its four 3-hyperedges) has a high structural overlap with the single 4-hyperedge of $G_{2}^{\left(4\right)}$. However, only NMI_cross_ is able to capture this similarity, giving a high value of 0.88. The other measures are unable to see any overlap among the hypergraphs except for the single edge (*7*, *8*), both giving low scores. Meanwhile, *G*_2_ and *G*_3_ are similar in that $G_{2}^{\left(3\right)}$ has a high structural overlap with $G_{3}^{\left(2\right)}$, and $G_{2}^{\left(4\right)}$ has maximal structural overlap with $G_{3}^{\left(4\right)}$. Since some of this overlap is now occurring at the same order ℓ=4—i.e., is intra-order similarity—both NMI_bulk_ and NMI_align_ are able to detect it, giving moderate scores. Looking at Eqs. 14 and 15, we can see that NMI_align_ will scale positively with both the number of overlapping hyperedges and the size of those hyperedges, contributing to fluctuations in this measure across the pairs of hypergraphs. Meanwhile, NMI_cross_ still gives the highest score, detecting both the intra- and cross-order similarity among the hypergraphs. Last, *G*_1_ and *G*_3_ are similar in that $G_{1}^{\left(2\right)}$ has a high structural overlap with $G_{3}^{\left(2\right)}$, and $G_{1}^{\left(3\right)}$ has a high structural overlap with $G_{3}^{\left(4\right)}$. In this case, since most of the hyperedges are of order ℓ=2, where *G*_1_ and *G*_3_ overlap, both NMI_bulk_ and NMI_align_ detect relatively high similarity values. Meanwhile, NMI_cross_ returns a perfect similarity score—$G_{1}^{\left(2\right)}$ and $G_{3}^{\left(2\right)}$ are identical, while $G_{1}^{\left(3\right)}$ is perfectly nested within $G_{3}^{\left(4\right)}$.

As discussed, NMI_cross_ can obtain its maximum value of 1 for nonisomorphic hypergraphs, so long as they are completely nested within each other. However, nestedness must occur on a layer-layer basis to obtain perfect similarity. Thus, for *G*_2_ and *G*_3_—for which $G_{3}^{\left(2\right)}$ is nested within the union of layers $G_{2}^{\left(2\right)}$ and $G_{2}^{\left(3\right)}$—we have a slight information penalty since $G_{3}^{\left(2\right)}$ is not perfectly nested in either $G_{2}^{\left(2\right)}$ or $G_{2}^{\left(3\right)}$ individually (if multiple layers of *G*_2_ could be used to transmit a single layer of *G*_3_, one would incur an additional information cost for specifying the combination of layers, as well as a potentially substantial computational cost for checking possible layer combinations).

Last, we remark that one can apply the idea of cross-order similarity to the different orders of the same hypergraph to capture redundancies and reduce the dimensionality of the system preserving its essential structural features (*62*).

Numerically, NMI_bulk_ and NMI_align_ can be computed quickly with a runtime linear in the number of hyperedges in *G*_1_ and *G*_2_ by using set overlaps. However, NMI_cross_ is more challenging to compute due to the computation of the overlap $E_{i\to j}^{\left(k\to \ell \right)}$ in Eq. 17 and the projected layer size $E_{i}^{\left(k\to \ell \right)}$ in Eq. 21. This is because direct projection of the layer $G_{i}^{\left(k\right)}$ to obtain $G_{i}^{\left(k\to \ell \right)}$ quickly becomes computationally intractable as k,ℓ become large. For example, when *k* = 30 and ℓ=15, each hyperedge in layer *k* has more than 100 million subtuples of size ℓ which we must project onto to obtain the unique hyperedges contributing to $G_{i}^{\left(k\to \ell \right)}$. In section S1, we describe a recursive algorithm to implement NMI_cross_ efficiently, allowing for the fast comparison of hypergraphs with millions of nodes and large hyperedge orders using our NMI measures.

In section S2, we describe how to extend our measures to quantify the shared information among arbitrary coarse-grainings of nodes between a pair of hypergraphs. These multiscale hypergraph NMI measures allow for capturing hypergraph similarity at the scale of interest, as well as adapting the measures to multigraphs.

## RESULTS

To illustrate the hypergraph similarity measures introduced above, we first examine systems with variable intra-order similarity using the NMI_bulk_ and NMI_align_ measures. We then move through the hierarchy of measures and study hypergraphs with variable cross-order similarity, showing that the NMI_cross_ measure—the most expressive and flexible measure developed in our hierarchy of measures—more adequately captures such similarity than NMI_align_. Last, we apply NMI_cross_ to three empirical hypergraphs representing collaboration patterns in physics, the film industry, and software development, to analyze the patterns that are revealed in these systems using our framework.

### Intra-order similarity

To control the level of intra-order similarity among pairs of hypergraphs, we generate an initial hypergraph *G*_1_ as a random hypergraph over *N* = 100 nodes in which each layer $G_{1}^{\left(\ell \right)}$ for ℓ∈{2,3,4,5,6} is generated with a fixed number of hyperedges $E_{1}^{\left(\ell \right)}$ chosen uniformly at random from all $\left(\begin{array}{c} N\\ \ell \end{array}\right)$ possibilities. We then generate a second hypergraph *G*_2_ by starting with a copy of *G*_1_ and perturbing the hyperedges in *G*_2_ according to a noise parameter ϵ∈[0,1]. For each value ϵ, we choose a fraction ϵ of *G*_2_’s hyperedges uniformly at random and replace each with a randomly chosen hyperedge of the same size. In this way, for ϵ=0, we have that *G*_1_ and *G*_2_ are identical, while at ϵ=1, they are both equivalent to independently generated random hypergraphs. We then compute both NMI_bulk_ and NMI_align_ as we continue to inject structural noise by increasing ϵ. As discussed in the “Hierarchy of hypergraph similarity measures” section, the NMI_align_ measure is able to correct for heterogeneous densities across layers, a feature observed in many real-world hypergraphs (*64*) and which is not accounted for in NMI_bulk_. Therefore, we vary the relative densities $\rho^{\left(\ell \right)}=E^{\left(\ell \right)}/\left(\begin{array}{c} N\\ \ell \end{array}\right)$ of the layers in the initial random hypergraph *G*_1_ to examine the resulting discrepancy in the two measures.

**Fig. 2.**
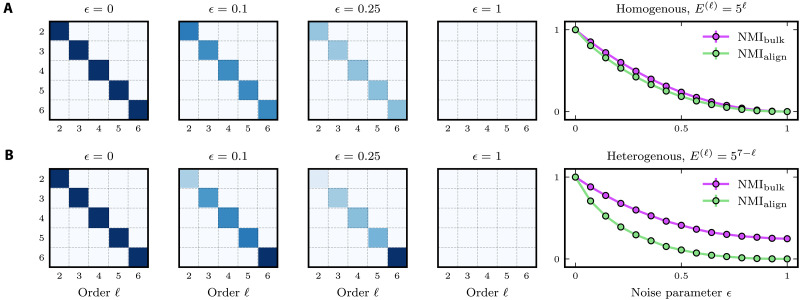
Information theory measures for intra-order hypergraph similarity. (**A**) Random hypergraphs with homogeneous layer densities. Order-order graph NMI values for the layers’ pairwise projections (left) show maximum shared structure at ϵ=0, which decreases uniformly as the layers are randomized. The intra-order hypergraph similarity measures NMI_bulk_ and NMI_align_ smoothly decrease with the noise ϵ, reaching zero in the regime of complete noise (right). Because of the homogeneous hyperedge densities across layers, both NMI measures give similar values. (**B**) Random hypergraphs with heterogeneous layer densities. Order-order similarities (left) indicate higher intra-order similarity for larger orders as noise is applied, due to the heterogeneous densities of the layers. In this case, on the right-hand plot, we see that NMI_bulk_ inflates the MI contributions for high ϵ, resulting in a non-negligible NMI value at ϵ=1. The NMI_align_ measure does not have this issue, vanishing in the high noise regime.

Figure 2 shows the results of these experiments. Each simulation was repeated 10 times, and the results were averaged, with error bars (vanishingly small for these experiments) indicating three SEs in the mean. In Fig. 2A, we plot the results for hypergraphs generated with $E^{\left(\ell \right)}=5^{\ell }$, to capture the exponential increase in the number of edges required to maintain a constant density $\rho^{\left(\ell \right)}$ as we increase ℓ. [Maintaining $\rho^{\left(\ell \right)}$ exactly while keeping a reasonable overall edge count results in too extreme a level of heterogeneity in the distribution of edge counts across layers, with $E^{\left(\ell \right)}\approx 0$ for all ℓ lower than the highest order.] The left four columns show the order-order similarity matrices computed using the graph NMI measure of (*47*) applied to the projections of each layer to the lower order of the two. We can see that the density of edges within each layer is unchanged by the noise and that the overlaps are quite homogeneous across the diagonal of the matrices due to the homogeneous layer densities. The off-diagonal entries nearly vanish in all cases, due to the overall sparsity of the hypergraphs and lack of nestedness among the layers. In the rightmost column, we show the results of applying our NMI measures. We see that both have a smooth decrease with the injected noise, as expected, reaching zero for ϵ=1. This illustrates that, for homogeneous edge densities across layers, both NMI formulations are capable of distinguishing meaningful hypergraph overlap from the spurious overlap expected due to the edge density.

However, this scenario—higher hyperedge counts for higher orders ℓ—is unlikely to be observed in practice. It is instead more realistic in practice to observe lower edge counts as we increase the order ℓ (*11*, *64*). In Fig. 2B, we show the same experiments for decreasing layer sizes $E^{\left(\ell \right)}=5^{7-\ell }$—this form ensures that the total number of edges, hence overall edge density $E/\left(2^{N}-N-1\right)$, is the same as in the previous experiments—which tell a different story. We can see that in this case, the similarity matrices to the left indicate a high level of heterogeneity in the similarities between layers. This results in very little change to NMI_align_ in the rightmost panel as we add noise to the system, with the curve approaching zero as before. However, the heterogeneous layer densities result in a severely inflated value of NMI_bulk_ in the high noise regime, meaning that it is no longer able to correct for the spurious overlap we see based on the densities of the layers. This suggests, as described in the “Hierarchy of hypergraph similarity measures” section, that the NMI_align_ measure is more appropriate for capturing intra-order similarity among hypergraphs with heterogeneous edge densities across layers.

### Cross-order similarity

While the intra-order comparisons made by NMI_bulk_ and NMI_align_ are relevant for cases where different orders of interaction are considered independent of one another, in many real-world applications, it is important to understand the structural similarity among hypergraphs while accounting for the nestedness of these interactions, as interactions that are nested may influence each other (*12*). It is therefore important to understand cross-order similarity among hypergraphs, which is the strength of the proposed NMI_cross_ measure.

To understand how NMI_cross_ performs compared to the intra-order similarity measure NMI_align_ when nestedness is perturbed, we design a third generative model, the block-nested hypergraph, which explicitly encodes dependencies between layers of different sizes across hypergraphs. In this model, we first generate “parent” layers ℓ∈{3,5,7} in *G*_1_ and *G*_2_ as random hypergraphs on *N* = 100 nodes with $E^{\left(\ell \right)}=N\left(\begin{array}{c} 7\\ \ell \end{array}\right)$ hyperedges for each ℓ. We then pick the layers ℓ=2,4, and 6 to be “child” layers, corresponding to the parent layers ℓ=3,5, and 7, respectively. We leave the parent layers unchanged and set each child layer $G_{j}^{\left(\ell \right)}$ in hypergraph *G_j_* to be the projection $G_{i}^{\left(k\right)}$ of its parent layer in the other hypergraph *G_i_*. This creates hypergraphs with perfect cross-order overlap among parent-child layer pairs across the hypergraphs. To vary the level of intra-order similarity, we keep some parent layers ℓ∈{3,5,7} identical across the two hypergraphs and allow others to be generated independently at random.

**Fig. 3.**
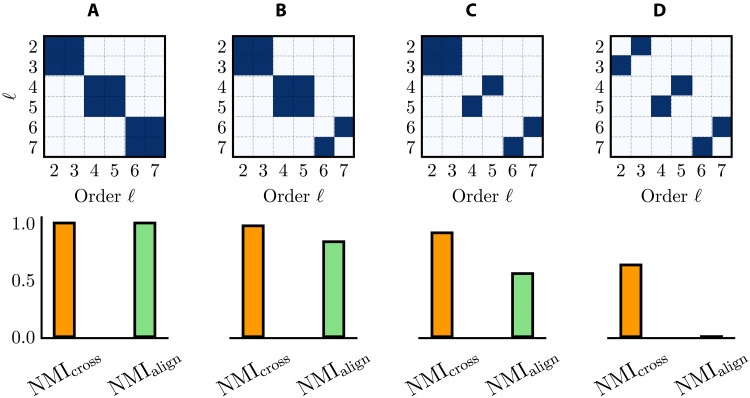
Information theory measures for cross-order hypergraph similarity. (**A**) Two initial random hypergraphs share the same layers ℓ=2,4, and 6, which are nested inside of ℓ=3,5, and 7, respectively. (**B**) Layers 6 and 7 are perturbed, causing their respective blocks to lose intra-order similarity. The intra-order measure NMI_align_ is thus reduced, while the cross-order measure NMI_cross_ changes negligibly. (**C**) Layers 4 and 5 are further perturbed, removing another nested block. Intra-order similarity is significantly reduced yet again. (**D**) Last, layers 2 and 3 are perturbed, dismantling all blocks and eliminating any similarity between layers of equal size. The intra-order score NMI_align_ approaches the minimum value of zero, while NMI_cross_ is still able to capture the structural similarity across different orders of interaction.

We show the results of applying NMI_align_ and NMI_cross_ to these synthetic hypergraph pairs in Fig. 3. In the top row, we plot the order-order similarity (as in Fig. 2) across the two hypergraphs for each experimental setting, and in the bottom row, we plot the two NMI measures as a bar chart. In Fig. 3A, we keep all parent layers ℓ∈{3,5,7} identical across the hypergraphs. In this case, both measures return a value of 1 as expected. In the system shown in Fig. 3B, the parent layer ℓ=7 is generated independently at random across the two hypergraphs. However, layer ℓ=6 of *G*_2_ is still generated as a child nested within layer ℓ=7 of *G*_1_, while layer ℓ=6 of *G*_1_ is the child of layer ℓ=7 of *G*_2_. Thus, generating the layers ℓ=7 independently destroys the intra-order similarity at ℓ=7, but the parent-child relationship still enforces overlap between layers ℓ=7 and ℓ=6 across the two hypergraphs. We can see that this perturbation results in nearly no detectable change in NMI_cross_ and a moderate decrease in NMI_align_.

In the system of Fig. 3C, we proceed from the configuration in Fig. 3B and generate layer ℓ=5 independently at random across hypergraphs. Again, by construction, layer ℓ=4 of *G*_2_ is generated to be nested within layer ℓ=5 of *G*_1_, and similarly ℓ=4 of *G*_1_ is nested within layer ℓ=5 of *G*_2_. In this case, we can see again that the cross-order measure NMI_cross_ is nearly unchanged, while NMI_align_ exhibits a notable decline to around 0.5. Last, in Fig. 3D, we fully destroy the intra-order similarity by allowing all parent layers to be generated independently while preserving the parent-child relationships across layers. Here, we can see that NMI_cross_ shows a modest drop, while NMI_align_ almost completely vanishes. Drops in NMI_cross_ are due to the transmission cost of the parent layers ℓ=3,5,7: Since these do not have any parent from which they can be transmitted cheaply, they must be transmitted from layers in the opposite hypergraph that are potentially uncorrelated, decreasing the NMI. In section S3, we further examine the three proposed NMI measures in various other models of hypergraphs with tunable nestedness, finding intuitive results that show little discrepancy for non-nested systems and support the usage of NMI_cross_ for nested systems.

### Applications to real-world systems

To illustrate the applicability of our information theoretic framework for hypergraph similarity, we study three empirical systems that are naturally represented as multiplex hypergraphs—systems consisting of multiple independent hypergraphs on the same set of nodes. We study collaboration networks from three different disciplines: physics (*65*), film (*66*), and software development (*67*–*69*). In each dataset, a hyperedge is formed among ℓ nodes (individuals) if these ℓ individuals contributed to the same paper/movie/repository (*70*, *71*). The hypergraphs in each multiplex system are organized by categorical metadata: subfields of physics in the American Physical Society (APS) dataset, movie genres in the Internet Movie Database (IMDb) corpus, and repository tags in the Rust open-source ecosystem. Further descriptions and summary statistics for these datasets can be found in section S4.

**Fig. 4.**
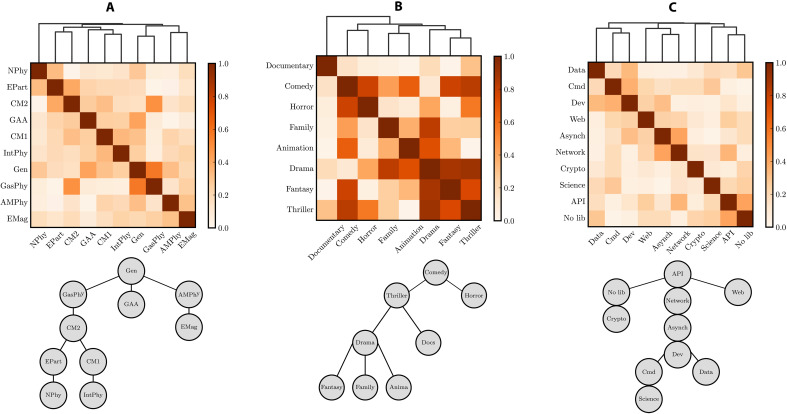
Hypergraph similarity for real-world systems. NMI matrices among all pairs of hypergraphs within real-world systems arising across various disciplines (top row), each accompanied by its corresponding minimum spanning tree using 1 − NMI as an edge weight. (**A**) Multiplex hypergraph of coauthorship among physics authors in different physics fields. (**B**) Multiplex hypergraph of coappearances among actors in different film genres. (**C**) Multiplex hypergraph of repository coediting among software development teams. For each system, the similarity among hypergraphs corresponding to qualitatively similar subjects (e.g., nuclear and elementary particle physics) tends to be higher. CM2, condensed matter 2; GAA, geophysics, astronomy, astrophysics; Emag, electromagnetism.

Figure 4 shows the results of applying the NMI_cross_ measure to all pairs of hypergraphs in each multiplex. In the top row, we show matrices containing these hypergraph NMI values, along with corresponding dendrograms constructed from hierarchical clustering using the Ward criterion. Below each similarity matrix, we plot a minimum spanning tree constructed using 1 − NMI_cross_ as edge weights. For each dataset, the NMI values between hypergraphs from qualitatively similar categories of interactions tend to be high, with less similarity assigned to disparate categories. For example, in the APS dataset, we see a high value of similarity between Nuclear (NPhy) and Elementary Particle physics (EPart), which in turn are dissimilar to Gas physics (GasPhy). Meanwhile, for the IMDb dataset, we observe high similarity among the thriller and drama genres, for which the genre boundary is often unclear. Meanwhile, we see very little similarity among documentaries and other genres due to the fundamentally different nature of acting in documentaries. Last, for open-source software collaborations, we see a high NMI between the hypergraphs corresponding to command line utilities (Cmd), development tools (Dev), and data structures (Data), which are functionally related via shared code and common use of custom data types. Section S4 shows the order-order similarity matrices among pairs of hypergraphs in each dataset, giving support to the findings discussed here. Also, in section S6, we show the runtime scaling of our measures on these empirical datasets.

In section S7, we explore a further application of our method to real hypergraph data, this time to detect anomalies in temporal higher-order interactions. In section S8, we then study the sensitivity of NMIcross for detecting structural overlaps that affect dynamical contagion processes on hypergraphs, connecting this measure of structural similarity to similarity in dynamical behaviors. Last, in section S9, we compare our measures with a simple Jaccard similarity baseline and alternative measures of structural similarity (*28*). These results, together with those in our synthetic tests, show the effectiveness of our hypergraph similarity measures for capturing meaningful structural overlap among hypergraphs in both real and controlled settings.

## DISCUSSION

Given the growth in complexity and dimensionality of relational datasets, quantifying the similarity between hypergraphs is an increasingly important challenge in network science. Existing approaches to this task tend to rely on ad hoc heuristics and/or tunable parameters, to which results are highly sensitive. Moreover, many of these methods fail to scale to large real-world hypergraphs with varying layers of higher-order interactions, with no prescription for correcting for spurious overlaps due to edge density. In this work, we introduce a principled, flexible framework for constructing MI measures between hypergraphs and construct a hierarchy of hypergraph similarity measures using this framework to highlight structural overlaps among hypergraphs at different scales and orders of interaction. Across a series of synthetic experiments, we showed that each measure behaves in an intuitive manner, highlighting structural similarity precisely in the way prescribed by the measure’s encoding scheme. In particular, our measure NMI_cross_ proved essential to capture the most general notion of hypergraph similarity, detecting structural correspondence both within and across layers of different orders. Meanwhile, extension to a multiscale measure was required for detecting similarity beyond the node level. We further demonstrated the practical value of our methods through applications to empirical multiplex hypergraphs from three distinct collaboration domains.

The proposed framework opens up several avenues for future exploration. We only explored a few different encodings in this paper—the bulk, align, and cross encodings, along with their corresponding multiscale variants. However, the NMI measure in Eq. 9 applies to any desired encoding, leaving open the possibility for more sophisticated models capturing more nuanced aspects of similarity among hypergraphs. For example, in (*47*), we explore a degree-corrected variant of the graph NMI to capture ego network–level similarities, a variant which would be possible to explore also for higher-order data. Moreover, one could explore compression via community structure (*72*) to more efficiently encode the hypergraphs being compared. Our method might also be extended to account for node or edge metadata, as well as temporal or multiplex hypergraph structure, to accommodate a wider variety of real-world data. Last, it may be possible to modify these measures to encode and cluster entire populations of more than two hypergraphs arising from longitudinal or cross-sectional studies, extending existing work for pairwise graphs (*40*, *73*).

There are also numerous important applications in which our framework might prove useful. In neuroscience, for example, higher-order network representations of neural activity also provide distinct structural signatures undetectable with standard pairwise methods (*74*, *75*). Our framework would allow for the comparison and clustering of higher-order brain networks across subjects and experimental conditions in such studies to reveal underlying regularities in functional connectivity.

Last, there are a few key limitations of the hierarchy of NMI measures we present here which, if addressed, can allow for the proposed NMI framework to be applied to a wider variety of systems. First, although the NMI framework in the “Hypergraph normalized MI framework” section applies to any pair of hypergraphs, the proposed hierarchy of NMI measures in this section apply only to node-aligned hypergraphs. This covers a wide range of cross-sectional and longitudinal network measurements but is not inclusive of all possible hypergraphs one may wish to compare. For hypergraphs with the same number of nodes but without alignment in their labels, one can, in principle, fix the node labels of *G*_1_ and sample the space of node labelings for *G*_2_ with simulated annealing or another Markov chain Monte Carlo technique to find the configuration that maximizes the NMI between the two hypergraphs. Meanwhile, for unaligned networks with different numbers of nodes, one could expand this scheme for node alignment by restricting the graph with more nodes to a subset of nodes equal in size to other hypergraph; finding the best alignment with simulated annealing as described above; and repeating these two steps until convergence of the node labels and NMI (alternatively, one could add nodes to the smaller of the two hypergraphs until the number of nodes is equal and then apply the stochastic matching without the need for selecting a subset of nodes). Both of these modifications would, however, incur a substantial additional computational expense to the otherwise highly efficient NMI measures presented here. In addition, while the mesoscale NMI we propose can be directly applied to compare hypergraphs with edge weights that are positive integers (see section S2), our measures do not yet account for hypergraphs with noninteger or negative edge weights. Such an extension could increase the compatibility of our framework with hypergraphs constructed from time series data, in which correlations may be both signed and continuous. Our work provides foundational tools for the principled comparison of higher-order network datasets, shedding light on the structural organization of empirical systems with nondyadic interactions.

## Supplementary Materials

### This PDF file includes:

Supplemetary Text Figs. S1 to S13 Tables S1 to S3 References
