# Supplementary material for: Information theory for hypergraph similarity
Source: Sci Adv. 2026 Jun 3;12(23):eaec5619. doi: 10.1126/sciadv.aec5619 (PMC13232591; doi:10.1126/sciadv.aec5619)
Supplement: Supplementary file 1 — Supplemetary Text Figs. S1 to S13 Tables S1 to S3 References [file sciadv.aec5619_sm.pdf]

Science Advances
AAAS

Supplementary Materials for

Information theory for hypergraph similarity

Helcio Felippe *et al.*

Corresponding author: Alec Kirkley, alec.w.kirkley@gmail.com; Federico Battiston, battistonf@ceu.edu

Sci. Adv. **12**, eaec5619 (2026)
DOI: 10.1126/sciadv.aec5619

This PDF file includes:

Supplemetary Text
Figs. S1 to S13
Tables S1 to S3
References

S1. EFFICIENT IMPLEMENTATION OF $\text{NMI}_{\text{cross}}$

To calculate $\text{NMI}_{\text{cross}}(G_1, G_2)$ numerically for hypergraphs G_1, G_2 with large hyperedge orders $\mathcal{L}$, the computational bottleneck lies in computing $E_{i\to j}^{(k\to\ell)}$ in Eq. (17) and $E_i^{(k\to\ell)}$ in Eq. (21). For $k, l \lesssim 10$, these two quantities can be computed by projecting $G_i^{(k)}$ to obtain $G_i^{(k\to\ell)}$, then computing the set intersection of $G_i^{(k\to\ell)}$ and $G_j^{(\ell)}$ to calculate $E_{i\to j}^{(k\to\ell)}$ and the size of $G_i^{(k\to\ell)}$ to calculate $E_i^{(k\to\ell)}$. However, for $k, l \gtrsim 10$, the direct projection of a k-tuple onto its $\binom{k}{\ell}$ subsets of size ℓ becomes computationally costly, effectively becoming intractable for $k, l \gtrsim 30$. Here we design a recursive counting scheme to determine $E_{i\to j}^{(k\to\ell)}$ and $E_i^{(k\to\ell)}$ directly without projection, allowing us to compute $\text{NMI}_{\text{cross}}(G_1, G_2)$ efficiently for hypergraphs with large hyperedges.

For large k, ℓ, we can compute $E_i^{(k\to\ell)}$ by iterating through the edges $G_i^{(k)} = \{e_1, \ldots, e_{E_i^{(k)}}\}$ in a fixed order, for each edge e_t checking its overlaps $o(e_t) = \{e_t \cap e_\tau : \tau < t\}$ with all previously checked edges. Then, we can compute the number of new projected tuples that e_t contributes to $E_i^{(k\to\ell)}$ as $\binom{k}{\ell} - E^{(o(e_t)\to\ell)}$, where $E^{(o(e_t)\to\ell)}$ is the number of unique subtuples of size ℓ within the set of overlapping tuples $o(e_t)$, which can be computed recursively using the same approach. Meanwhile, the overlap $E_{i\to j}^{(k\cap\ell)}$ can be efficiently computed by iterating over the hyperedges $e_t \in G_i^{(k)}$ and incrementing $E_{i\to j}^{(k\cap\ell)}$ for each edge $e_s \in G_j^{(\ell)}$ that fully overlaps with the larger tuple e_t, removing e_s from $G^{(\ell)}$ after the comparison if it overlapped with e_t.

The computations of $E_i^{(k\to\ell)}$ and $E_{i\to j}^{(k\to\ell)}$ using the above counting methods incur a total computational complexity of roughly $O\left[(E_i^{(k)})^2 + E_i^{(k)} E_j^{(\ell)}\right]$ rather than the $O\left(\binom{k}{\ell} E_i^{(k)}\right)$ complexity using the projection $k \to \ell$. Thus, it becomes more efficient to use these algorithms for $\binom{k}{\ell} \gtrsim E_i^{(k)}, E_j^{(\ell)}$. Since the conditional entropy is computed for all layer pairs $k \geq \ell$ in hypergraphs i, j respectively to determine $k_i^{(\ell)}$ in Eq. (19), the overall runtime complexity for computing the $\text{NMI}_{\text{cross}}(G_1, G_2)$ is at most roughly $O(E^2 L^2)$, with E the typical number of hyperedges in any given layer ℓ. In practice, we find that this measure easily scales to hypergraphs with millions of nodes and hundreds of layers.

S2. MULTISCALE HYPERGRAPH SIMILARITY

The encodings of Sec. II C explore various ways to compute similarity among hypergraphs at the node-level, meaning that two hyperedges in different hypergraphs only contribute shared information to the NMI if they have exactly the same node set or one is a subset of the other. But in some applications it is more relevant to assess the similarity among two systems at a coarser scale, beyond the node-level. For example, when examining whether two hypergraphs have statistically similar modular structure—which, crucially, does not necessarily mean overlap among their individual hyperedges—the measures of Sec. II C fail to capture the desired aspects of similarity. To consider an extreme example, a pair of hypergraphs generated from the exact same ensemble of sparse hypergraphs with identical node community partitions (76) will have almost no overlap according to the measures of Sec. II C, and thus will have NMI scores close to zero. These networks are statistically identical at the level of their modular structure, by construction, but one must "zoom out" beyond the node-scale to the community-scale to capture it.

Following a similar line of reasoning as in (47), we can generalize the NMI measures of Sec. II C to a family of *multiscale* NMI measures that assess similarity among the pair of hypergraphs G_1, G_2 with respect to a shared partition $\boldsymbol{b}$ of their nodes, where b_n is the label of node $n \in \{1, \ldots, N\}$. The partition $\boldsymbol{b}$ of the nodes can be obtained either exogenously from node metadata or endogenously based on network structure, for example a community detection algorithm. In this case, we do not want to compare the similarity among G_1 and G_2 directly, but rather coarse-grained versions $\tilde{G}_1^{(\boldsymbol{b})}$ and $\tilde{G}_2^{(\boldsymbol{b})}$ of these hypergraphs in which all nodes of the same group label in $\boldsymbol{b}$ are treated as identical. The object $\tilde{G}_i^{(\boldsymbol{b})}$ can be mathematically treated as a *multiset* in which each ℓ-tuple (edge) $(n_1, \ldots, n_\ell)$ in layer ℓ is converted to an ℓ-tuple $(b_{n_1}, \ldots, b_{n_\ell})$ of partition labels—sorted to correctly account for duplicates—and identical tuples may be repeated. Letting B be the number of unique node labels (e.g. groups) in $\boldsymbol{b}$, and the scale of individual nodes to be $O(N^{-1})$, the multiscale similarity measures we propose assess similarity between G_1 and G_2 at the scale $O(B^{-1})$. Thus, when we have few groups, $B \sim O(1)$, our multiscale NMI measures assesses hypergraph similarity at the macro-scale $O(1)$. On the other hand, when $B \sim O(N)$ and we have an extensive number of small groups of nodes, our multiscale NMI measures assesses hypergraph similarity at the node-scale $O(N^{-1})$ just as with the measures in Sec. II C. In the extreme case $B = N$, our multiscale measures can be used to extend the measures of Sec. II C to multigraphs or integer-weighted graphs, as these can be represented as multisets on N nodes.

The multiscale NMI measures are largely the same structurally as the standard hypergraph NMI measures we present. However, in the multiscale case there are a different number of unique (sorted) hyperedges of size ℓ that can be constructed from the B unique node labels in $\boldsymbol{b}$, which will impact the entropy and conditional entropy measures' configuration spaces.

In order to adapt our NMI measures to compare the multisets $\tilde{G}_1^{(\boldsymbol{b})}$ and $\tilde{G}_2^{(\boldsymbol{b})}$, we need to utilize the *multiset coefficient*

$$\left(\!\!\binom{n}{k}\!\!\right) = \binom{n+k-1}{k}, \tag{S1}$$

which is the number of unique multisets of size k that can be constructed from a set of n unique items (51). Additionally, it will be important to extend the concept of intersection to multisets, which can be done by defining the intersection $\cap_m$ of the multisets M_1 and M_2 as

$$M_1 \cap_m M_2 = \sum_{x \in M_1, M_2} \min(M_1(x), M_2(x)), \tag{S2}$$

where $M_i(x)$ is the number of times element x occurs in multiset M_i. This reduces to the standard set intersection when $M_i(x) \in \{0, 1\}$.

For the multiscale bulk NMI measure, the entropy can be modified as follows. There are $\left(\!\binom{B}{\ell}\!\right) = \binom{B+\ell-1}{\ell}$ unique undirected hyperedges of size ℓ that can be constructed in layer ℓ. Therefore, there are

$$\sum_{\ell=2}^{N} \binom{B+\ell-1}{\ell} = \sum_{\ell=0}^{N} \binom{(B-1)+\ell}{(B-1)} - B - 1 = \binom{B+N}{B} - B - 1 \tag{S3}$$

ways to construct hyperedges of size up to N using the B unique node labels, from which we must choose a multiset of size E_i to specify $\tilde{G}_i^{(\boldsymbol{b})}$. The appropriate modification of Eq. (11) is then

$$\mathrm{H}_{\mathrm{bulk}}^{(\boldsymbol{b})}(G_i) = \log \left(\!\!\binom{\binom{B+N}{B} - B - 1}{E_i}\!\!\right). \tag{S4}$$

The multiscale bulk conditional entropy measure can then be adapted as follows. There are $E_i^{(\ell)}$ hyperedges in layer ℓ of $\tilde{G}_i^{(\boldsymbol{b})}$, of which we must choose

$$E_{12}^{(\boldsymbol{b})} = |\tilde{G}_1^{(\boldsymbol{b})} \cap_m \tilde{G}_2^{(\boldsymbol{b})}| \tag{S5}$$

hyperedges to specify the hyperedges that overlap with $\tilde{G}_j^{(\boldsymbol{b})}$. We then must specify a multiset of size $E_j - E_{12}^{(\boldsymbol{b})}$ from the $\binom{B+N}{B} - B - 1$ possible hyperedges to specify the remaining hyperedges of $\tilde{G}_j^{(\boldsymbol{b})}$. The appropriate modification of the conditional entropy is thus

$$\mathrm{H}_{\mathrm{bulk}}^{(\boldsymbol{b})}(G_j|G_i) = \log \binom{E_i}{E_{12}^{(\boldsymbol{b})}} \left(\!\!\binom{\binom{B+N}{B} - B - 1}{E_j - E_{12}^{(\boldsymbol{b})}}\!\!\right). \tag{S6}$$

As we cannot in general say that $\mathrm{H}_{\mathrm{bulk}}^{(\boldsymbol{b})}(G_j|G_i) \leq \mathrm{H}_{\mathrm{bulk}}^{(\boldsymbol{b})}(G_j)$, to ensure non-negativity of the NMI we enforce the entropy as an upper cutoff to the conditional entropy so that $\mathrm{H}_{\mathrm{bulk}}^{(\boldsymbol{b})}(G_j|G_i) \to \min\left[\mathrm{H}_{\mathrm{bulk}}^{(\boldsymbol{b})}(G_j|G_i),\, \mathrm{H}_{\mathrm{bulk}}^{(\boldsymbol{b})}(G_j)\right]$. This is equivalent to saying that G_j will be transmitted by itself if G_i does not aid in its transmission, and is a result of the expression for the conditional entropy being only an upper bound for this multiscale case. Equations (S4) and (S6) can then be plugged into Eq. (9) to find the multiscale variant $\mathrm{NMI}_{\mathrm{bulk}}^{(\boldsymbol{b})}$ of $\mathrm{NMI}_{\mathrm{bulk}}$, which is bounded in $[0, 1]$.

Using the same line of logic, we can compute $\mathrm{NMI}_{\mathrm{align}}^{(\boldsymbol{b})}$ and $\mathrm{NMI}_{\mathrm{cross}}^{(\boldsymbol{b})}$ using the following adaptations of the entropy and conditional entropy measures of Sec. II C:

$$\mathrm{H}_{\mathrm{align}}^{(\boldsymbol{b})}(G_i) = \sum_{\ell \in \mathcal{L}} \log \left(\!\!\binom{\left(\!\binom{B}{\ell}\!\right)}{E_i^{(\ell)}}\!\!\right), \tag{S7}$$

$$\mathrm{H}_{\mathrm{align}}^{(\boldsymbol{b})}(G_j|G_i) = \sum_{\ell \in \mathcal{L}} \log \binom{E_i^{(\ell)}}{E_{12}^{(\ell,\boldsymbol{b})}} \left(\!\!\binom{\left(\!\binom{B}{\ell}\!\right)}{E_j^{(\ell)} - E_{12}^{(\ell,\boldsymbol{b})}}\!\!\right), \tag{S8}$$

$$\mathrm{H}_{\mathrm{cross}}^{(\boldsymbol{b})}(G_i) = \sum_{\ell \in \mathcal{L}} \log \left(\!\!\binom{\left(\!\binom{B}{\ell}\!\right)}{E_i^{(\ell)}}\!\!\right), \tag{S9}$$

$$\mathrm{H}_{\mathrm{cross}}^{(\boldsymbol{b})}(G_j|G_i) = \sum_{\ell \in \mathcal{L}} \log \binom{E_i^{(k_i^{(\ell,\boldsymbol{b})} \to \ell)}}{E_{i \to j}^{(k_i^{(\ell,\boldsymbol{b})} \to \ell, \boldsymbol{b})}} \left(\!\!\binom{\left(\!\binom{B}{\ell}\!\right)}{E_j^{(\ell)} - E_{i \to j}^{(k_i^{(\ell,\boldsymbol{b})} \to \ell, \boldsymbol{b})}}\!\!\right), \tag{S10}$$

where

$$E_{ij}^{(\ell,\boldsymbol{b})} = |\tilde{G}_i^{(\boldsymbol{b},\ell)} \cap_m \tilde{G}_j^{(\boldsymbol{b},\ell)}|, \tag{S11}$$

$$E_{i\to j}^{(k\to\ell,\boldsymbol{b})} = |\tilde{G}_i^{(\boldsymbol{b},k\to\ell)} \cap_m \tilde{G}_j^{(\boldsymbol{b},\ell)}| \tag{S12}$$

are the appropriately modified overlap measures, with $\tilde{G}_i^{(\boldsymbol{b},\ell)}$ the layer of hyperedges of size ℓ in $\tilde{G}_i^{(\boldsymbol{b})}$, and $\tilde{G}_i^{(\boldsymbol{b},k\to\ell)}$ the projection of the layer $\tilde{G}_i^{(\boldsymbol{b},k)}$ onto hyperedges of size ℓ. We have also defined

$$k_i^{(\ell,\boldsymbol{b})} = \arg\min_{k\geq\ell}\left\{\log\binom{E_i^{(k\to\ell)}}{E_{i\to j}^{(k\to\ell,\boldsymbol{b})}}\left(\!\!\binom{\left(\!\binom{B}{\ell}\!\right)}{E_j^{(\ell)} - E_{i\to j}^{(k\to\ell,\boldsymbol{b})}}\!\!\right)\right\} \tag{S13}$$

analogously to Eq. (19).

It is worth noting that this mesoscale measure can be directly applied as a means to assess similarity among weighted hypergraphs, in the common case that the edges are positive integers (such as counts or frequencies in temporal systems). In this case, we can treat the hypergraphs as multi-hypergraphs with an edge e of weight $w(e)$ corresponding to $w(e)$ independent hyperedges on the tuple of nodes e. We then set the partition $\boldsymbol{b}$ for each hypergraph to be the partition into N groups of size 1, such that the labels of the nodes are unchanged. This allows the encodings to properly account for the multi-edges when assessing similarity, by using the multiset combinatorics described above.

In Fig. S1(a) we show an illustration of the regular and mesoscale variants of $\text{NMI}_{\text{cross}}$ between two small example hypergraphs, with communities $\boldsymbol{b}$ indicated in yellow and pink. When ignoring the node partition $\boldsymbol{b}$, the NMI is quite low (0.2), as there is little structural overlap among the hypergraphs at the node-level. However, when we apply $\text{NMI}_{\text{cross}}^{(\boldsymbol{b})}$, we find maximum similarity due to identical coarse-grained representations $\tilde{G}_i^{(\boldsymbol{b})}$ at the mesoscale, which are not captured by the regular NMI measure.

We then run simulations using synthetic hypergraph pairs G_1, G_2 on $N = 1000$ nodes, tuning the level of planted community structure and level of similarity in their underlying node partitions $\boldsymbol{b}^{(1)}, \boldsymbol{b}^{(2)}$, which we set to have $B = 50$ groups. We fix the layer sizes to $E^{(\ell)} = 2^{12-\ell}$ for $\ell \in \{2, \ldots, 10\}$ and generate each ℓ-hyperedge through repetition of the following process $E^{(\ell)}$ times:

1. Choose a group $r \in \{1, \ldots, B\}$ at random to form the majority affiliation of a new hyperedge.
2. Generate group affiliations for each of the remaining $\ell - 1$ nodes by picking group r with probability p and another community label $s \neq r$ uniformly at random from the remaining labels with probability $1 - p$.
3. For each community label in the hyperedge, pick a node from that community uniformly at random, without replacement.

This process results in random hypergraphs in which the expected fraction of nodes belonging to the majority community in each hyperedge is p. In this way, the individual hyperedges are independent and randomized across G_1, G_2, so we would expect NMI values near zero for the three original similarity measures discussed in Sec. II C. However, the mesoscale NMI measure $\text{NMI}_{\text{cross}}^{(\boldsymbol{b}^{(1)})}$ should be able to detect the similarity among the generated hypergraphs at the level of the planted modular structure. We expect that as the graphs G_1, G_2 become less modular—i.e., the level of community strength p decreases—the mesoscale similarity should decrease.

We can also vary the extent to which the modular structure overlaps across the two hypergraphs. For this, we take the partition $b^{(1)}$ used to generate G_1 and shuffle pairs of elements to form the partition $\boldsymbol{b}^{(2)}$ which is used to generate G_2. We use a parameter $\rho_{\boldsymbol{b}}$ to tune this shuffling, with $\rho_{\boldsymbol{b}} = 0$ corresponding to no shuffling and $\rho_{\boldsymbol{b}} = 1$ correspond to swapping $N/2$ pairs of elements, so that all community labels have been perturbed.

In Fig. S1(b) we show the results of these experiments. On the left we plot the mesoscale NMI versus the level of partition noise $\rho_{\boldsymbol{b}}$ for hypergraph pairs with various levels of community strength p. Markers are again averages over ten trials, with error bars representing three standard errors in the mean. We can see that the mesoscale NMI measure attributes maximum similarity for maximal community strength $p = 1$ when the partitions are not shuffled ($\rho_{\boldsymbol{b}} = 0$). We can also see that it attributes a similarity of nearly zero for all $\rho_{\boldsymbol{b}}$ when there is very weak community structure ($p = 0$). As we decrease the strength of community structure p, we interpolate between these two regimes, with smooth decreases in similarity for greater levels of partition noise in all cases. On the right of Fig. S1(b), we allow the community strengths p_1 and p_2 to be different between the two hypergraphs for $\rho_{\boldsymbol{b}} = 0$, finding that mesoscale similarity is detected at high levels until around $p_i \approx 0.25$. This may be indicating a "detectability transition" (77) in the planted community structure, in which the hyperedges are no longer correlated with the underlying shared node partition $\boldsymbol{b}$ in any meaningful way, resulting in a vanishing mesoscale NMI.

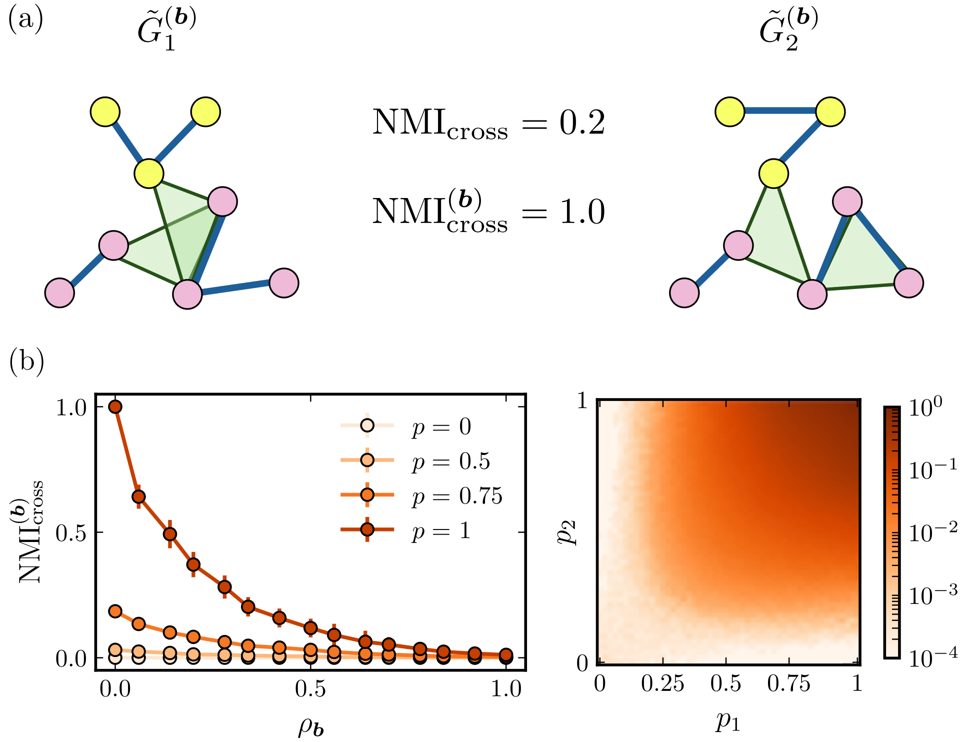

FIG. S1. **Mesoscale similarity for hypergraphs.** (a) $\mathrm{NMI_{cross}}$ and its mesoscale variant $\mathrm{NMI}^{(\boldsymbol{b})}_{\mathrm{cross}}$ for two small example networks on $N = 8$ nodes, with the partition $\boldsymbol{b}$ dividing the nodes into $B = 2$ groups indicated in yellow and pink. While the mesoscale measure is able to detect perfect similarity among the coarse-grained hypergraphs $\tilde{G}_1^{(\boldsymbol{b})}$ and $\tilde{G}_2^{(\boldsymbol{b})}$, the standard NMI variant detects a low level of similarity at the node-level. (b) Mesoscale NMI for pairs of random clustered hypergraphs generated with an average fraction p of nodes belonging to the same group. As we increase the level of noise $\rho_{\boldsymbol{b}}$ between the two hypergraphs' underlying node partitions, the mesoscale NMI smoothly decreases, with stronger levels of community structure p resulting in a more severe decline in the NMI (left). When both hypergraphs are generated from the same underlying node partition ($\rho_{\boldsymbol{b}} = 0$) with different community strengths p_1, p_2, we see that greater levels of community structure result in greater levels of shared information among the hypergraphs, with $p_1 = p_2 = 1$ giving maximum similarity.

S3. SIMILARITY OF HYPERGRAPHS WITH TUNABLE NESTEDNESS

Here, we extend the analysis of intra- and cross-order similarity for more complex models of synthetic hypergraphs with tunable levels of nestedness. In particular, we compute the $\mathrm{NMI}_{\mathrm{bulk}}$, $\mathrm{NMI}_{\mathrm{align}}$, and $\mathrm{NMI}_{\mathrm{cross}}$ of $N = 100$ node hypergraphs under various levels of noise ϵ. We describe the models and experiments below, followed by Fig. S2 showing the results. At the end of this section (Fig. S3), we also illustrate in detail the randomization procedure for a paradigmatic example of synthetic hypergraph, i.e. the block-nested hypergraph model used to highlight cross-order similarity in the absence of intra-order similarity. Similar procedures are employed to generate the other synthetic structures.

- Fully nested hypergraphs with identical noise. We initialize two hypergraphs G_1 and G_2 over the same set of $N = 100$ nodes. We then generate, independently at random, interactions of order $\ell = 7$. Interactions of lower orders $\ell \in \{2, 3, 4, 5, 6\}$ are generated by selecting all tuples of nodes which are subsets of the tuples encoding interactions of order 7. The layers of interaction are assigned to G_1 and G_2, making them identical fully nested hypergraphs. We add noise to both G_1 and G_2 at the same noise level ϵ, rewiring all orders of interaction identically in each hypergraph. In this case, layers of the same order are kept identical across hypergraphs while layers of different orders become uncorrelated within the hypergraphs. All three NMI scores indicate perfect similarity, as expected.
- Fully nested hypergraphs with independent noise. Same as the previous model, but the layers of hypergraphs G_1 and G_2 are independently rewired. All NMI measures start at the maximum similarity, but smoothly decay to zero since layers of all sizes become uncorrelated across the hypergraphs.
- 2-block-nested hypergraphs. We generate, independently at random, interactions of order $\ell = 4$. Interactions of order 2 and 3 are generated by selecting all tuples of nodes which are subsets of the tuples of order 4. Analogously, we generate independently at random interactions of order 7, and generate orders 5 and 6 by selecting tuples which are all subsets of interactions at layer 7. The layers are then independently attacked such that their shared block-structure is destroyed. All NMI scores smoothly decrease with ϵ.
- 3-block-nested hypergraphs. Same procedure as previous model, but with a three-block architecture instead: layer 3 generates layer 2; layer 5 generates layer 4; and layer 7 generates layer 6. Graphs are independently rewired and all scores smoothly decrease with ϵ as before.
- Intertwined hypergraphs. We generate independently at random interactions of order 3, 5, and 7 in hypergraph G_i. We then take the corresponding subsets of these layers and assign them, respectively, to layers 2, 4, and 6 of G_j. We then attack both hypergraphs independently. Since the hypergraphs never shared intra-order similarity, the $\mathrm{NMI}_{\mathrm{bulk}}$ and $\mathrm{NMI}_{\mathrm{align}}$ assigns zero similarity throughout the whole noise process, whereas $\mathrm{NMI}_{\mathrm{cross}}$ is able to detect the shared structure embedded across different layers of the hypergraphs.
- Anti-block-nested hypergraphs. We initialize two "reference" 2-block-nested hypergraphs H_A, H_B, where layers 4 and 7 generate, respectively, layers 2, 3, and 5, 6 in both H_A and H_B. We then assign the block layer $\ell \in \{2, 3, 4\}$ from H_A to G_1, and $\ell \in \{5, 6, 7\}$ from H_B to G_1. Analogously, we take the block layer $\ell \in \{2, 3, 4\}$ from H_B to G_2, and $\ell \in \{5, 6, 7\}$ from H_A to G_2. Both graphs are independently attacked. Only the $\mathrm{NMI}_{\mathrm{cross}}$ measure is able to detect shared similarity prior to the full rewiring at $\epsilon = 1$.

Throughout the experiments, the density of hyperedges is kept meaningful across all layers of interactions, in the sense that the size of layer ℓ is set at $E^{(\ell)} = E^{(\ell_{\max})}\binom{\ell_{\max}}{\ell}$ with a choice of $E^{(\ell_{\max})} \geq 100$.

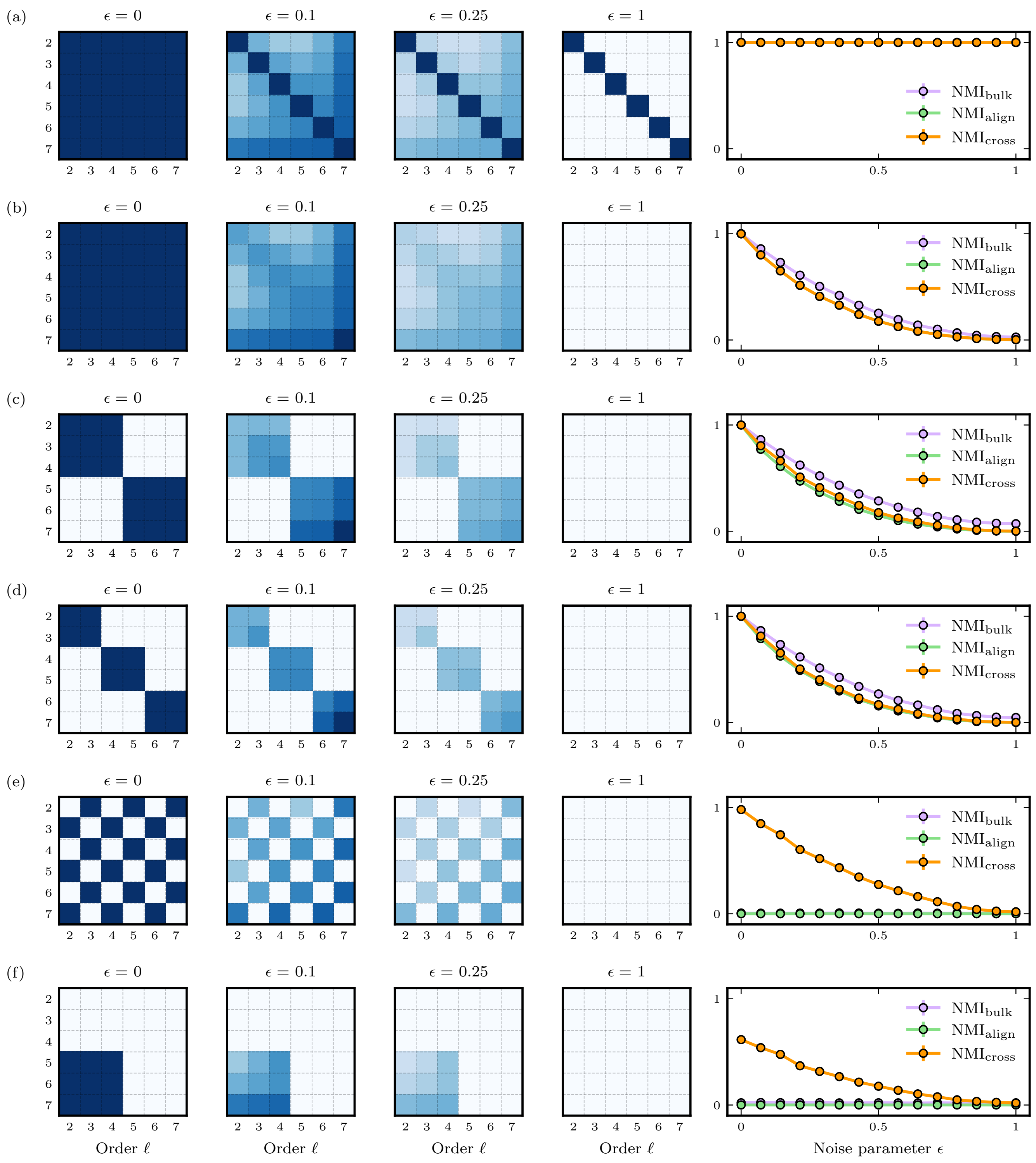

FIG. S2. **Similarity scores against noise parameter for hypergraphs with tunable nestedness.** (a) Fully nested hypergraphs dependently attacked. (b) Fully nested hypergraphs independently attacked. (c) 2-block-nested hypergraphs. (d) 3-block-nested hypergraphs. (e) Intertwined hypergraphs. (f) Anti-block-nested hypergraph.

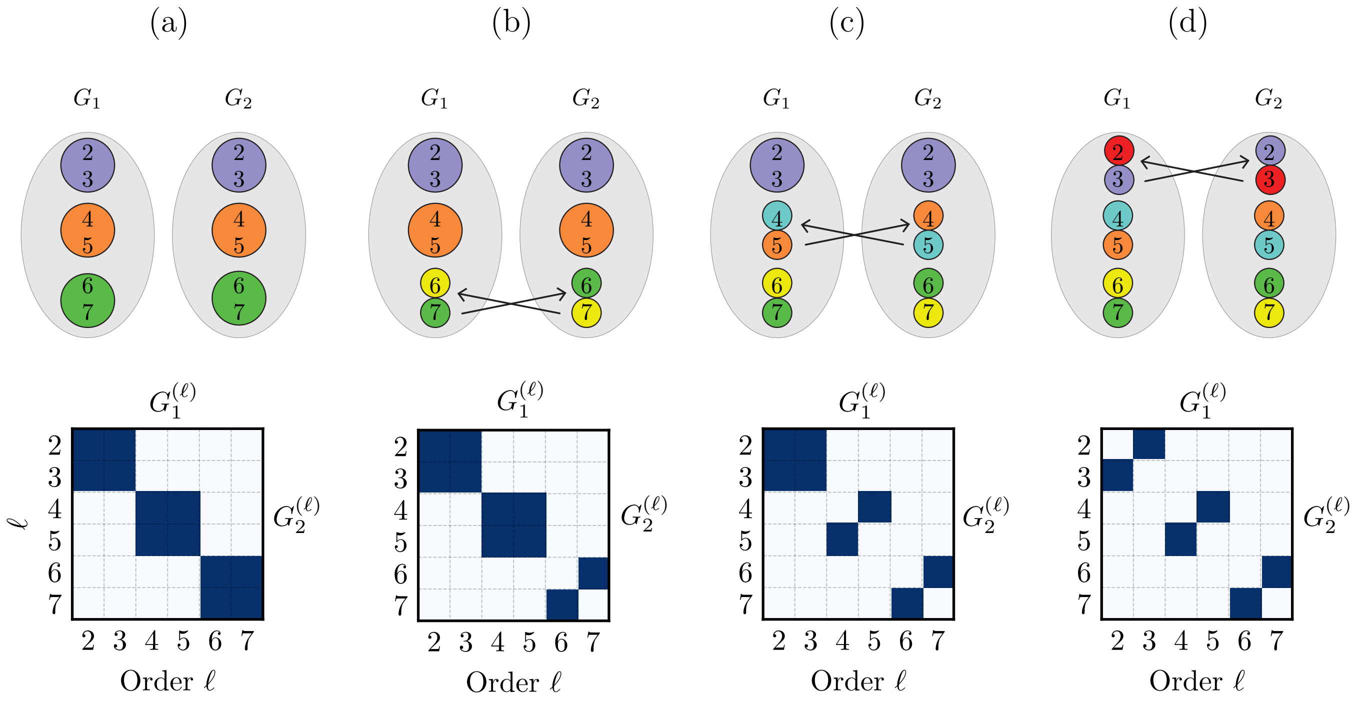

FIG. S3. **Randomization procedure of the block-nested hypergraph model.** (a) We generate "parent" layers $\ell \in \{3,5,7\}$ in both G_1 and G_2 and populate "child" layers $\ell' \in \{2,4,6\}$ as the projections of $G_i^{(\ell=\ell'+1)}$ in the *other* hypergraph $G_j^{(\ell')}$. That is, layers 2, 4, and 6 of G_2 are nested in layers 3, 5, and 7 of G_1 (and vice-versa in this initial setting). (b) We generate parent layer $\ell = 7$ independently at random in G_1, G_2, then generate the child layers $\ell = 6$ in G_2, G_1 respectively as projections of these parent layers at $\ell = 7$. Arrows point from parent layer to child layer. (c) We start with the system in (b) and generate parent layer $\ell = 5$ independently at random in G_1, G_2, then generate the child layers $\ell = 4$ in G_2, G_1 respectively as projections of these parent layers at $\ell = 5$. (d) We start with the system in (c) and generate parent layer $\ell = 3$ independently at random in G_1, G_2, generating child layers as before. Intra-order similarity between hypergraphs is destroyed and only cross-order similarity remains, as seen in the heatmaps of the lower row (reproduced from the main text).

S4. EMPIRICAL MULTIPLEX HYPERGRAPHS

Here we present summary statistics and pre-processing details for the three multiplex hypergraph datasets shown in the main text, representing scientific collaboration (APS physics fields (65)), movie co-appearances (IMDb movie genres (66)), and software development teams (Rust Github repositories (67–69)).

The APS multiplex dataset (65) contains ten layers representing ten physics fields according to the Physics and Astronomy Classification Scheme (PACS) of the American Physical Society (APS). Each layer-field is a hypergraph in which actors are nodes connected via hyperedges representing a paper published in that particular field. For instance, a paper with three authors in Nuclear Physics is a hyperedge of size three in the corresponding layer "NPhy". Layers vary in terms of number of nodes N, total number of hyperedges edges E, and maximum order of interaction $\ell_{\max}$. For example, the condensed matter subfields (CM1 and CM2) tend to have papers with only a few authors, while the Elementary Particles (EPart) layer has some papers with thousands of authors. See Table S1 for further details.

The IMDb multiplex dataset contains eight layers representing eight movie genres according to the Internet Movie Database (IMDd). Each layer is a hypergraph in which actors are nodes connected via hyperedges representing their co-appearance within a movie of the corresponding genre (see Table S2).

The Github multiplex contains ten layers representing ten categories from the Rust Github repositories. Each layer-repository is a hypergraph in which users are nodes connected via hyperedges representing collaboration on a project in the corresponding category (see Table S3).

For each dataset, we considered only nodes that co-appeared in at least two different layers, allowing for the presence of cross-order overlap. As described in the main text, we then computed the $\mathrm{NMI}_{\mathrm{cross}}$ score between each layer of the multiplex in order to assess the similarity of physics fields, movie genres, and repository categories (Fig. 4 in the main text). Figure S4 illustrates our preprocessing and analysis of the empirical multiplex hypergraphs. Below we show the results of computing the pairwise similarity for the different orders of interaction $\ell = 2, \ldots, 10$ in the same manner as in Figs. 1-2. Figure S5 shows the similarity between orders of interaction for all combinations of physics fields. Most PAC pairs show high similarity scores only for lower-order interactions, with the exception of a few pairs such as the condensed matter fields and nuclear and interdisciplinary physics (NPhy and IntPhy). Similar results are shown for the IMDb and Github datasets in Figs. S6 and S7, respectively.

Finally, we highlight that throughout our empirical analysis we took the intersection of the node sets in G_1 and G_2 as their common node set of size N. This allowed for comparisons only with respect to the nodes that actively participate in both hypergraphs, which is preferable if some nodes are naturally constrained to only exist in one of the two hypergraphs. For example, in the scientific co-authorship hypergraphs, we focus on interdisciplinary authors that publish papers in multiple disciplines (each discipline being an independent hypergraph). In this case, since a large portion of authors have short academic careers confined to a single discipline, and the frequency of attrition is discipline-dependent, it is more sensible to compare hypergraphs based on the authors that are active in multiple disciplines to understand structural similarities in collaboration patterns. An alternative option for hypergraphs of non-identical node sets is to use the union of the node sets in G_1 and G_2 as the shared node set of size N, which requires adding isolated nodes to one or both node sets until they match. This approach is preferable when comparing systems in which the absence of nodes provides important evidence of structural dissimilarity, since it provides an increasingly strong penalty on the NMI as the node sets of G_1, G_2 overlap less. Either choice of preprocessing is compatible with the specific encodings we present in Sec. II C.

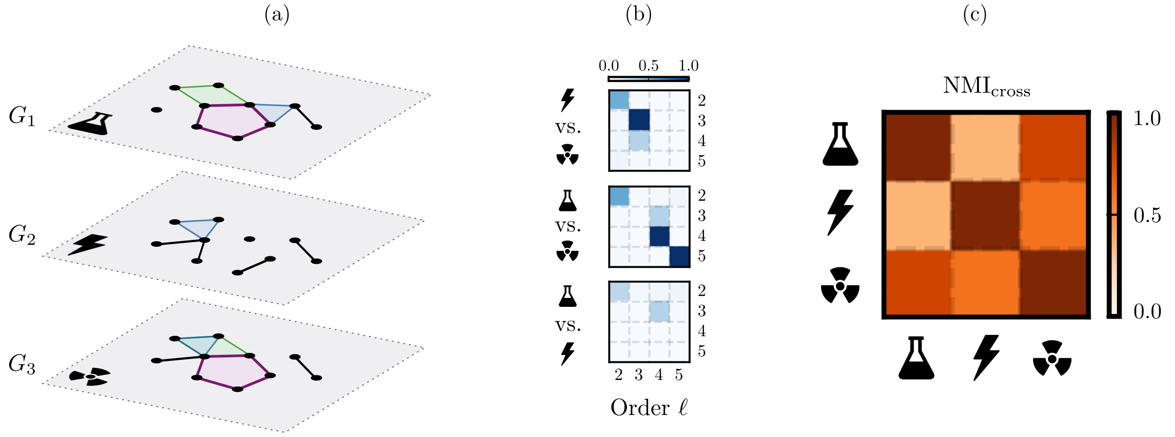

FIG. S4. **Preprocessing and analysis of empirical datasets.** (a) Diagram of multiplex hypergraph containing three layers G_1, G_2, and G_3. (b) Order-order similarity heatmaps of the multiplex layers. (c) $\mathrm{NMI}_{\mathrm{cross}}$ between layers of the multiplex.

**PACS**	N	E	$\ell_{\max}$	$N^{(\ell\leq 10)}$	$E^{(\ell\leq 10)}$
General (Gen)	87712	48751	2926	74360	48077
Elementary Particles (EPart)	49920	18913	3047	19550	15256
Nuclear Physics (NPhy)	41335	14407	2895	14444	10285
Atomic and Molecular Physics (AMPhy)	36414	15599	76	32258	14892
Electromagnetism (EMag)	62335	37447	146	57502	36238
Physics of Gases (GasPhy)	12693	4031	409	9182	3554
Condensed Matter: Thermal Properties (CM1)	83351	40315	65	77596	38913
Condensed Matter: Optical Properties (CM2)	91267	51890	131	84001	49063
Interdisciplinary Physics (IntPhy)	71801	28823	627	65663	28065
Geophysics, Astronomy, and Astrophysics (GAA)	55975	14393	2921	29903	13542

TABLE S1. **Statistics of the APS multiplex hypergraph.**

**Genre**	N	E	$\ell_{\max}$	$N^{(\ell\leq 10)}$	$E^{(\ell\leq 10)}$
Comedy	58432	7992	313	15317	3190
Animation	8322	1372	100	3103	741
Family	21787	2465	313	6028	1168
Fantasy	21366	1925	313	5020	818
Drama	71894	10957	224	20755	4615
Thriller	49407	5905	158	12865	2369
Horror	30084	3173	95	8422	1359
Documentary	3570	482	112	1316	286

TABLE S2. **Statistics of the IMDb multiplex hypergraph.**

**Repository**	N	E	$\ell_{\max}$	$N^{(\ell\leq 10)}$	$E^{(\ell\leq 10)}$
API bindings (API)	1384	276	197	669	243
Asynchronous (Asynch)	964	35	50	612	216
Command line utilities (Cmd)	1216	275	113	719	253
Cryptography (Crypto)	925	25	76	460	205
Data structures (Data)	889	22	92	504	200
Development tools (Dev)	1594	370	76	846	326
Network programming (Network)	1090	266	64	714	250
No standard library (No lib)	1255	381	92	627	329
Science	701	41	197	364	133
Web programming (Web)	1003	236	64	620	219

TABLE S3. **Statistics of the Rust GitHub multiplex hypergraph.**

S5. SIMILARITY OF EMPIRICAL MULTIPLEX HYPERGRAPHS

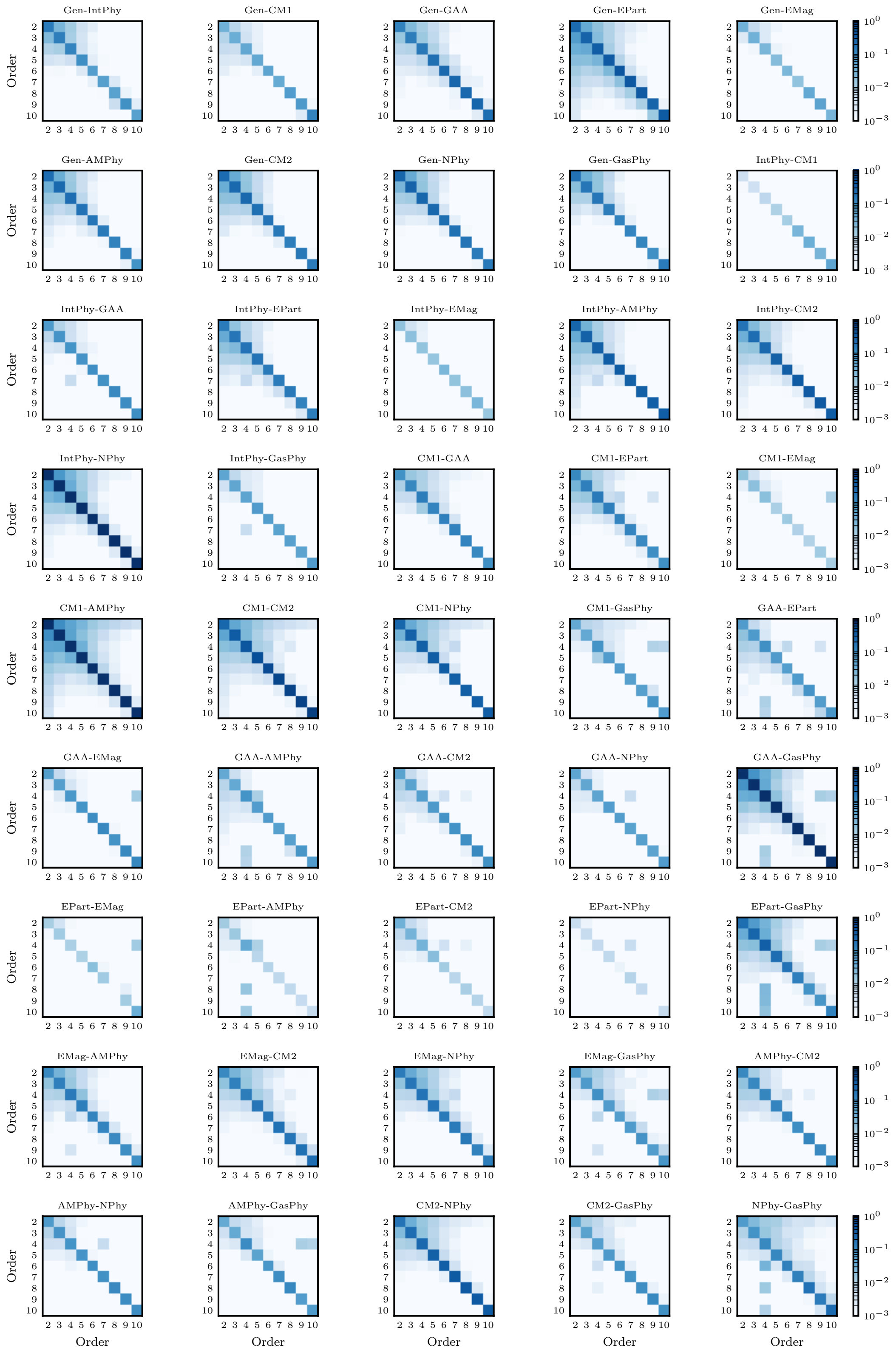

FIG. S5. **Order-order similarity matrices of the APS physics fields dataset.**

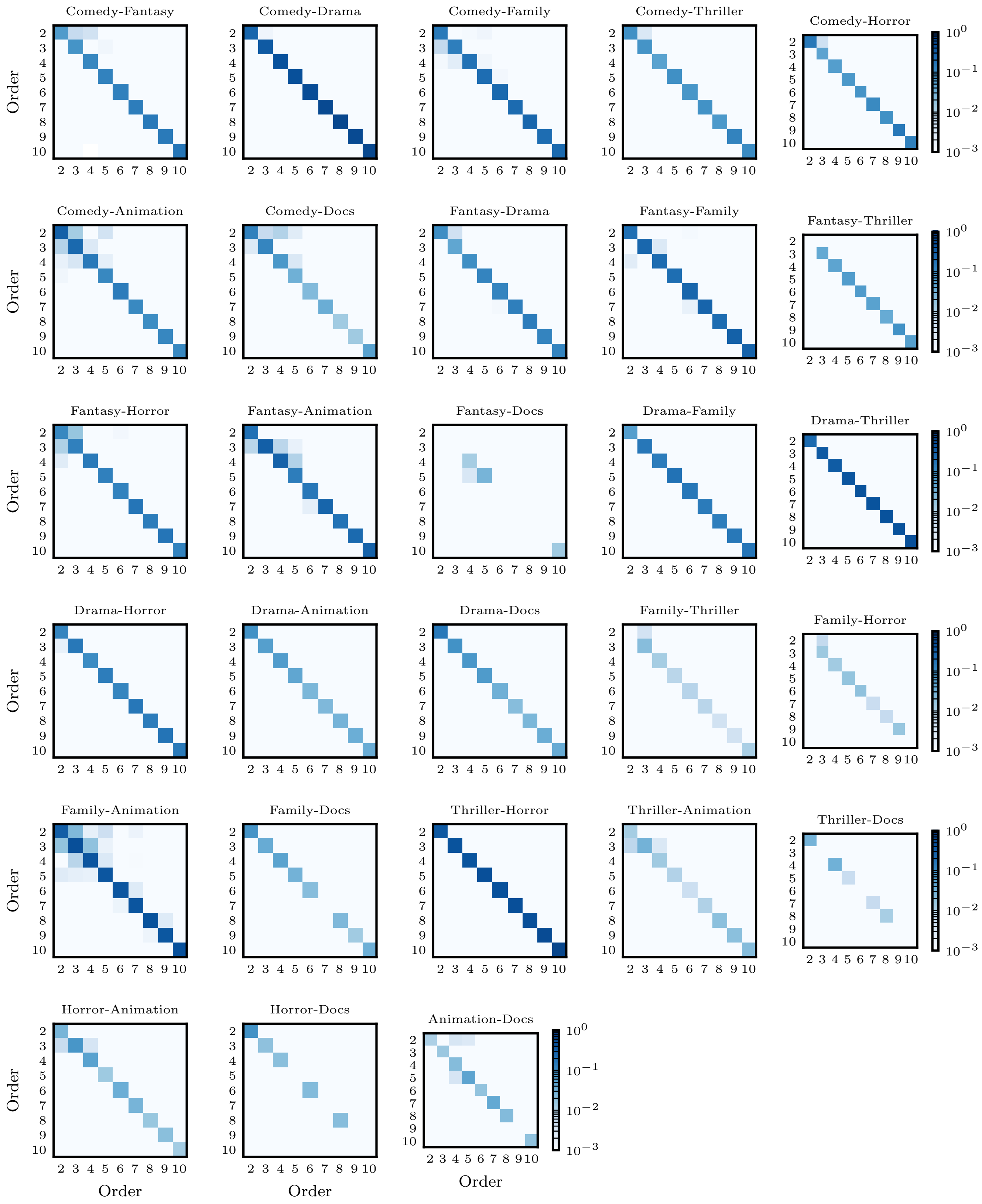

FIG. S6. **Order-order similarity matrices of the IMBd movie genres dataset.**

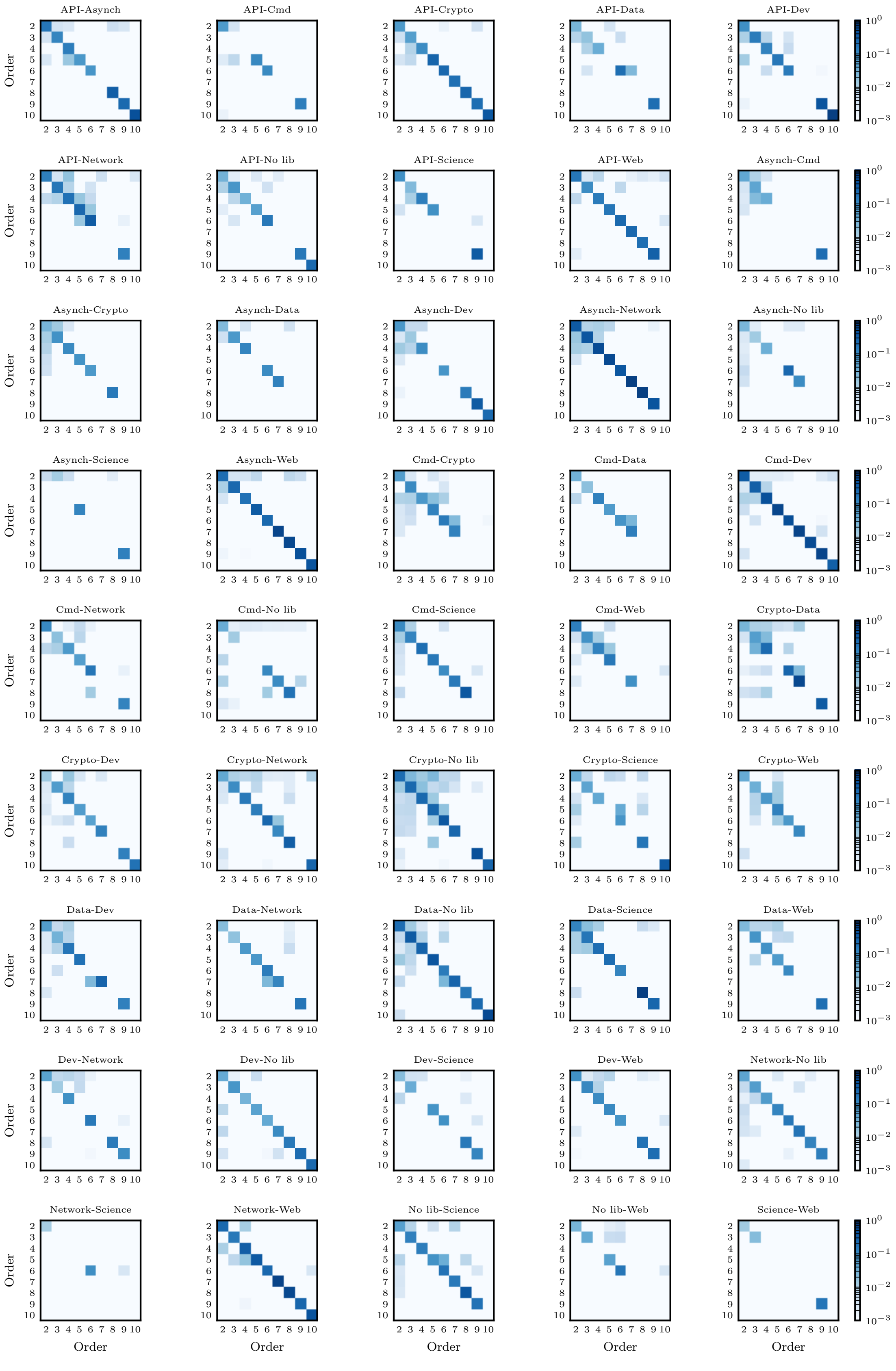

FIG. S7. **Order-order similarity matrices of the Rust Github repository dataset.**

S6. RUNTIME SCALING ON EMPIRICAL MULTIPLEX DATASETS

We compute the average runtime required, per pair of hypergraphs $\{G_i, G_j\}$, to compute the similarity values $\text{NMI}_{\text{cross}}(G_i, G_j)$ used in the experiments of Fig. 4. For each multiplex dataset, we examine how this runtime scales with the maximum layer order $\ell_{\max}$ included for the analysis, which gives estimates of the empirical runtime scaling behavior of our measure.

In Fig. S8 we show the results of these experiments for the three multiplex datasets. In row (a) we plot the results for all layers, while in row (b) we zoom in on the range $\ell_{\max} \in [2, 10]$. We find that, as expected, the runtime scaling is roughly quadratic in $\ell_{\max}$ for smaller values, in which all layers are occupied by hyperedges in most networks. We see slight deviations due to the number of edges in each layer—the hypothetical $O(\ell_{\max}^2)$ scaling of of SI Section S1 will only occur when all layers have an identical number of hyperedges. However, for very large maximum order $\ell_{\max}$, we find that the runtime starts to level off. This is because the layers are much more sparsely occupied—in many cases, empty—for higher ℓ.

Notably, the runtimes of NMI_{bulk} and $\text{NMI}_{\text{align}}$ are negligible on all the empirical hypergraphs, due to not considering cross-layer contributions which require either explicit projection or recursive counting of nested overlaps. These experiments give a more realistic idea of how the proposed measures scale with the size of empirical hypergraphs, complementing the theoretical scaling results of SI Section S1.

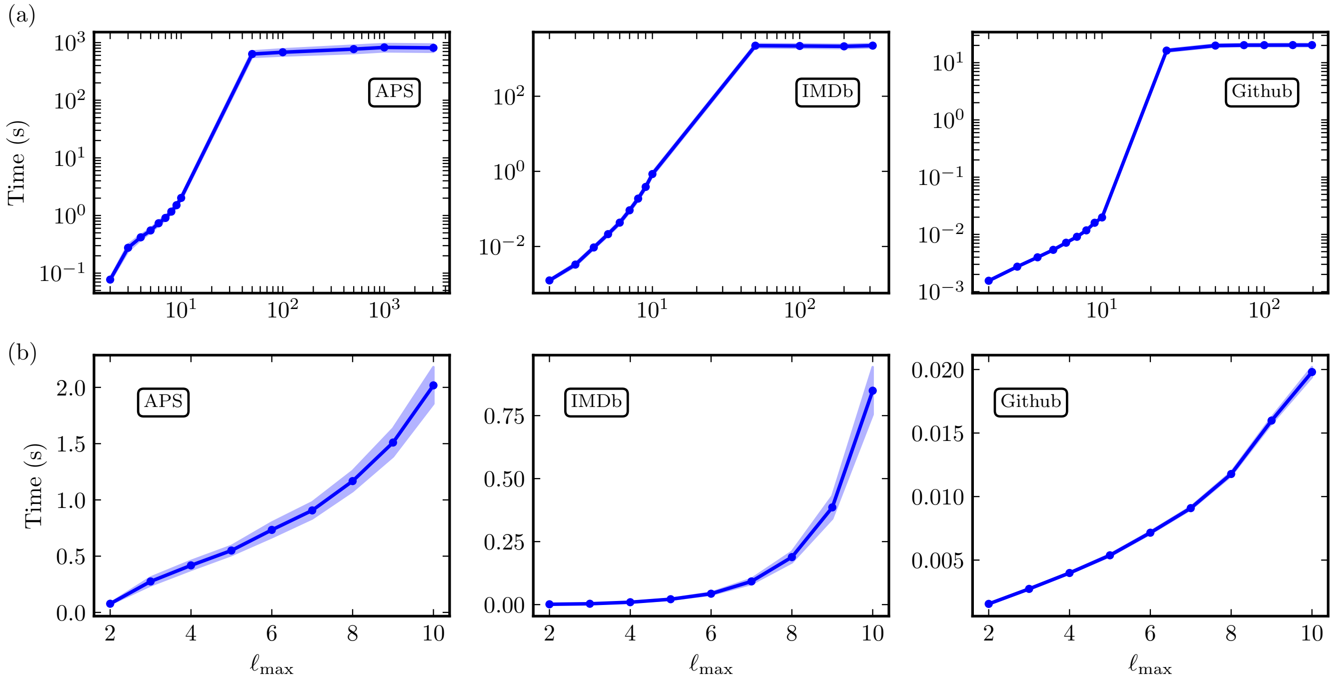

FIG. S8. **Runtime scaling in real multiplex data.**

S7. DETECTING ANOMALIES IN TEMPORAL HYPERGRAPH STREAMS

In longitudinal studies of social behavior in humans and other animals, one often looks for changepoints, anomalies, or other notable features in the temporal interaction dynamics, which are intrinsically higher-order in nature (64, 78). Applying the hypergraph similarity framework presented here to the temporal hypergraph snapshots of these systems allows for the detection of meaningful structural variability across time in such applications, which is not possible using standard graph similarity measures when groups vary in size over time (79).

Here we explore an application of our method for detecting anomalies in the Enron email dataset (80), which is naturally represented as a temporal hypergraph in which nodes are email addresses and each hyperedge represents the sender and all receivers of a particular email (81). Hyperedges are timestamped according to the time the email was sent, allowing for the construction of hypergraph snapshots for different time periods. For these analyses, we bin the hyperedges into thirty-day periods to capture month-month fluctuations in email activity, but the general conclusions we find persist under different binnings. The emails took place over a period of roughly 45 months in the late 1990s to early 2000s, during which the Enron corporation was involved in one of the largest accounting scandals in history. A number of works have aimed to understand the structure of these emails from the perspective of pairwise graphs (82, 83) and hypergraphs (81).

In Fig. S9(a) we show the dissimilarity $1-\text{NMI}(G_t, G_{t+1})$ among the emails from month t to month $t+1$, for all months t in the dataset. One curve shows the results obtained by computing the NMI using the pairwise projection of the hypergraph at time t and the graph NMI measure of (47), while the other curve shows the result of computing the NMI using the $\text{NMI}_{\text{cross}}$ measure we propose here. We also identify outliers in each time series using the crude (but widely used) interquartile range (IQR) method, in which any data point that exceeds the third quartile by more than 1.5 IQRs is considered a high outlier. Such high outliers in this case—i.e., anomalously high dissimilarity values—may correspond to abrupt shifts in the network structure of the emails, signifying an organizational change. These anomalies are highlighted as circular markers.

We can see that the time series constructed using the pairwise and hypergraph similarity measures share some underlying fluctuations but are largely uncorrelated, with only the pairwise series having anomalies according to the IQR method. This is because the pairwise measure does not capture the nested structure of the interactions, causing it to underestimate similarity in instances where hyperedges merge and split up over time. We also find qualitatively different autocorrelation structure among the two series: the hypergraph NMI time series has moderate to high positive autocorrelation for lags up to five months ($\{\rho(1), \rho(2), \rho(3), \rho(4), \rho(5)\} = \{0.49, 0.24, 0.40, 0.44, 0.35\}$), while the pairwise NMI time series only has positive autocorrelation for a lag of one month ($\{\rho(1), \rho(2), \rho(3), \rho(4), \rho(5)\} = \{0.31, -0.03, -0.09, -0.12, -0.09\}$). In Fig. S9(b) we plot the time series values as a scatterplot, which shows the lack of correlation among the series constructed using the pairwise and hypergraph NMI measures. The Pearson and Spearman correlation coefficients between the two series are -0.03 and 0.01 respectively.

These results provide an example of how, by enabling the detection of more nuanced aspects of similarity among datasets consisting of higher-order interactions, the proposed hypergraph similarity framework can provide qualitatively different conclusions in real-world application scenarios.

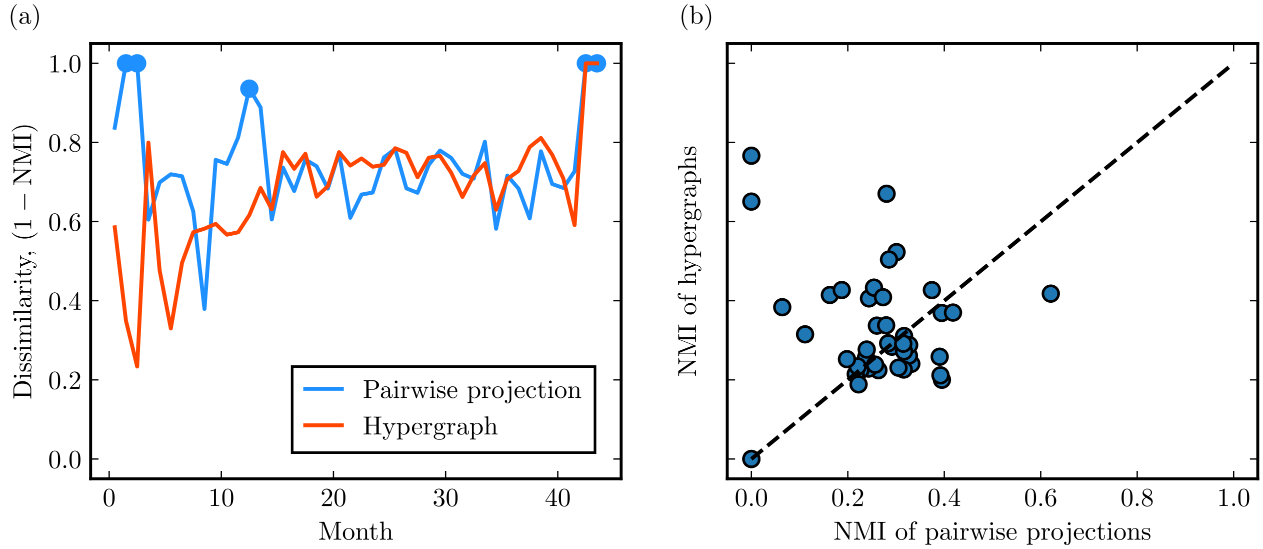

FIG. S9. **Detecting anomalies in temporal graph data.** (a) Month-to-month dissimilarity $1 - \mathrm{NMI}(G_t, G_{t+1})$ computed using the graph similarity (47) among the pairwise projections (blue) as well as the hypergraph similarity ($\mathrm{NMI}_{\mathrm{cross}}$, red), for the Enron email dataset (81). (b) Scatterplot of the time series values produced by both methods, with Pearson and Spearman correlation values of -0.04 and 0.01 respectively.

S8. HYPERGRAPH SIMILARITY AND CONTAGION DYNAMICS

Here we investigate quantitatively how structural similarity between two hypergraphs, as measured by our $\mathrm{NMI}_{\mathrm{cross}}$ measure, is linked to the outcome of a dynamical process running on the two systems. To perform such an analysis in a systematic way, we construct a fully nested hypergraph G — which will be used as our reference hypergraph — and progressively perturb its higher–order structure to create a second hypergraph G'. The perturbation consists of randomizing interactions independently across orders with probability $\epsilon \in [0, 1]$, where $\epsilon = 0$ corresponds to the original nested configurati and $\epsilon = 1$ corresponds to a fully randomized structure. Importantly, during this process we preserve the degree and hyper-degree distributions so that only the organization of interactions changes while the local connectivity statistics remain fixed. We consider regular hypergraphs with $N = 900$ nodes and a maximum number of layers $\ell_{\max} = 3$, the number of pairwise interactions and triplets fixed to $E^{(2)} = 4050$ and $E^{(3)} = 600$, respectively. The degree and hyper-degree distributions are also fixed at $k_1 = 9$ and $k_2 = 2$, such that these distributions remain unchanged throughout the entire perturbation procedure.

We then simulate a higher–order SIS contagion process (16) on the hypergraphs G, G' and study how the stationary prevalence state ρ^* varies as a function of the infectivity parameter λ_1. For each level of structural perturbation ϵ we estimate the epidemic threshold λ_1^* and compute the hyperaph similarity $\mathrm{NMI}_{\mathrm{cross}}(G, G')$. We can observe in Fig. S10(a) that the onset of the epidemic depends strongly on the structural organization of interactions: hypergraphs that are closer to the original nested configuration of G exhibit an earlier transition, while increasingly randomized structures delay the epidemic onset.

In Fig. S10(b) we plot the critical value λ_1^* as a function of similarity with the initial nested hypergraph. The results show a clear decreasing trend, indicating that as similarity to the original nested structure decreases, the epidemic threshold systematically shifts to larger values signaling a slower onset of contagion. This provides direct evidence that the proposed similarity measure captures structural features that are dynamically relevant for processes unfolding on higher–order networks. Our findings are in agreement with previous work which has shown that continuously perturbing structuraligher-order features can affect the onset of collective behavior in higher–order dynamical processes models (84).

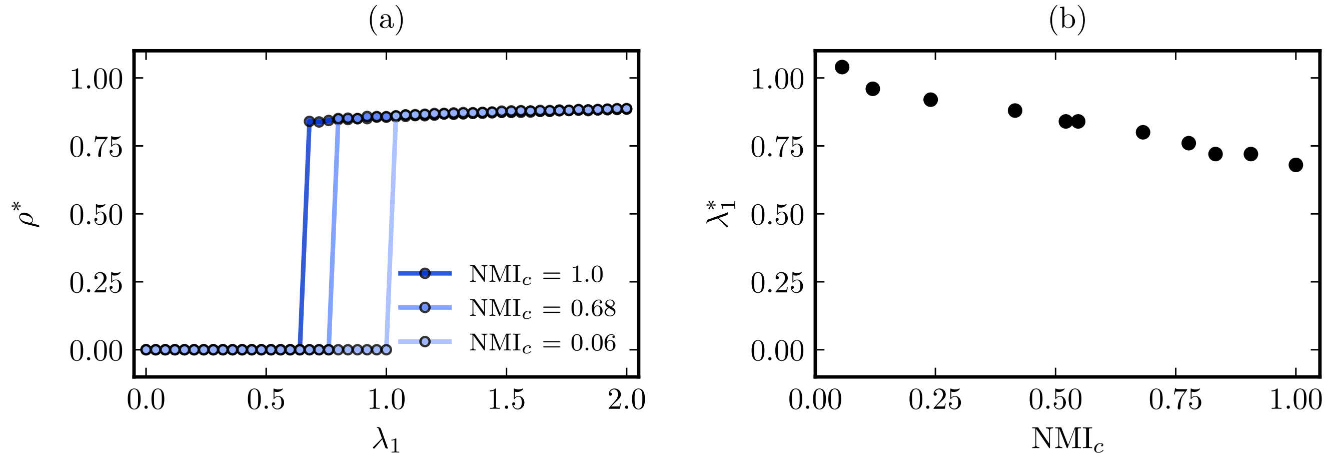

FIG. S10. **Epidemic onset and hypergraph similarity.** (a) Stationary prevalence ρ^* versus infectivity λ_1 for different structural perturbations of a nested hypergraph G. The cross-order NMI between G and its perturbed variant G' for different levels of perturbation noise are shown. (b) Epidemic threshold λ_1^* for the perturbed graph G' as a function of its NMI with G.

S9. COMPARISON WITH BASELINE AND EXISTING MEASURES

Here we compare our measures with a simple baseline and other recently proposed hypergraph similarity measures using the same synthetic tests as in the main text. A natural baseline for comparison is to take the average Jaccard similarity for the best hyperedge matching between the two hypergraphs G_1, G_2, thus

$$s(G_1,G_2) = \frac{1}{2|G_1|}\sum_{e\in G_1} \max_{e'\in G_2}\left\{\frac{|e\cap e'|}{|e\cup e'|}\right\} + \frac{1}{2|G_2|}\sum_{e\in G_2} \max_{e'\in G_1}\left\{\frac{|e\cap e'|}{|e\cup e'|}\right\} . \tag{S14}$$

This measure satisfies $s(G_1, G_2) = 1$ if and only if $G_1 = G_2$, and will decrease as the hyperedge sets become more dissimilar in hyperedge-level overlap, having equal contributions from each hypergraph to the similarity score. In Fig. S11, we reproduce Fig. 2 of the main text using this measure, finding that when compared to $\mathrm{NMI}_{\mathrm{bulk}}$ and $\mathrm{NMI}_{\mathrm{align}}$, this average Jaccard similarity measure has a similar smooth decrease with the noise level. However, it severely inflates the similarity of uncorrelated hypergraphs ($\epsilon = 1$) which have structural overlap purely due to chance. The NMI measures naturally correct for this since structural overlaps that do not greatly exceed those expected by chance will fail to provide any compression when considering the shared information among the hyperedge sets. As discussed in the main text, the NMI-align measure is more effective than NMI-bulk for accounting for this baseline level of overlap in the presence of heterogeneous layer densities, hence the gap in the curves in panel (b).

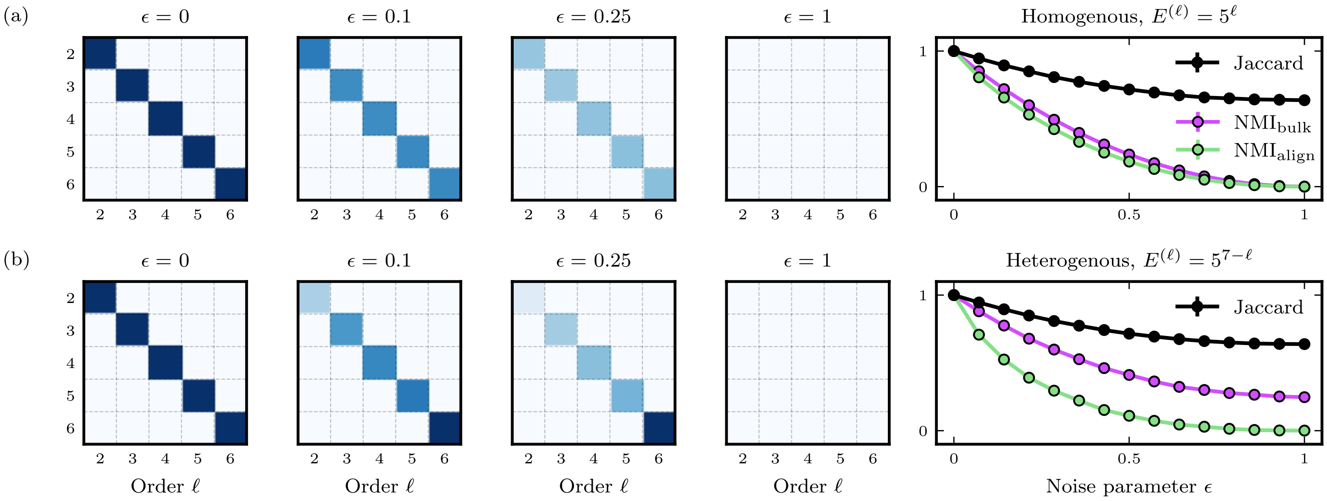

FIG. S11. **Comparison of NMI measures (bulk and align) against the Jaccard similarity index averaged over the best hyperedge matching.** The Jaccard similarity severely inflates the similarity value for uncorrelated hypergraphs, due to spurious hyperedge overlaps attributable to the hyperedge densities.

We also perform the same experiments with the two hypergraph dissimilarity measures proposed in (28). The first measure, Hyper NetSimile (HNS), is a generalization of the NetSimile measure (85) wherein graph similarity is regarded as the distance between signature vectors associated to multiple network descriptors. After considering generalized features such as hyper-degree, hyper-clustering coefficient, and so on, the HNS is defined as the Canberra distance between signature vectors $\boldsymbol{v}_1$ and $\boldsymbol{v}_2$ of hypergraphs G_1 and G_2, respectively,

$$\mathrm{HNS}(G_1,G_2) = d_{\mathrm{Canberra}}(\boldsymbol{v}_1,\boldsymbol{v}_2) = \frac{1}{V}\sum_{j=1}^{V}\frac{|v_1^j - v_2^j|}{|v_1^j|+|v_2^j|} , \tag{S15}$$

where the distance is normalized by $V = |\boldsymbol{v}_i|$. The second dissimilarity measure, Hyperedge Portrait Divergence (HPD), is the Jensen-Shannon divergence between distributions $P(m, n, l, k)$ associated to the number of hyperedges of size m having k hyperedges of size n at a distance l, such that $l = 1$ if at least one node is shared by the two hyperedges. In Fig. S12 we show the results of the *complement* of HNS and HPD (that is, $1 - \mathrm{HNS}$ and $1 - \mathrm{HPD}$, resp.) for the same experiment of Fig. 2 of the main text. Notably, for all noise parameters ϵ the dissimilarity measures are unable to distinguish the two random hypergraphs, regardless of their hyperedge layer density. This is because similarity with these two measures is assessed at a global level based on structural statistics rather than at a local level based on node IDs, which has the benefit of not requiring node alignment but is unable to distinguish the actual node sets that form the hyperedges so is

not suited for analyzing node-aligned systems. In this experiment, as noise is added, the hypergraphs continue to have similar structural statistics despite the decreasing overlap in the node identities within their hyperedges. This results in the persistent high similarity values we see in these two measures.

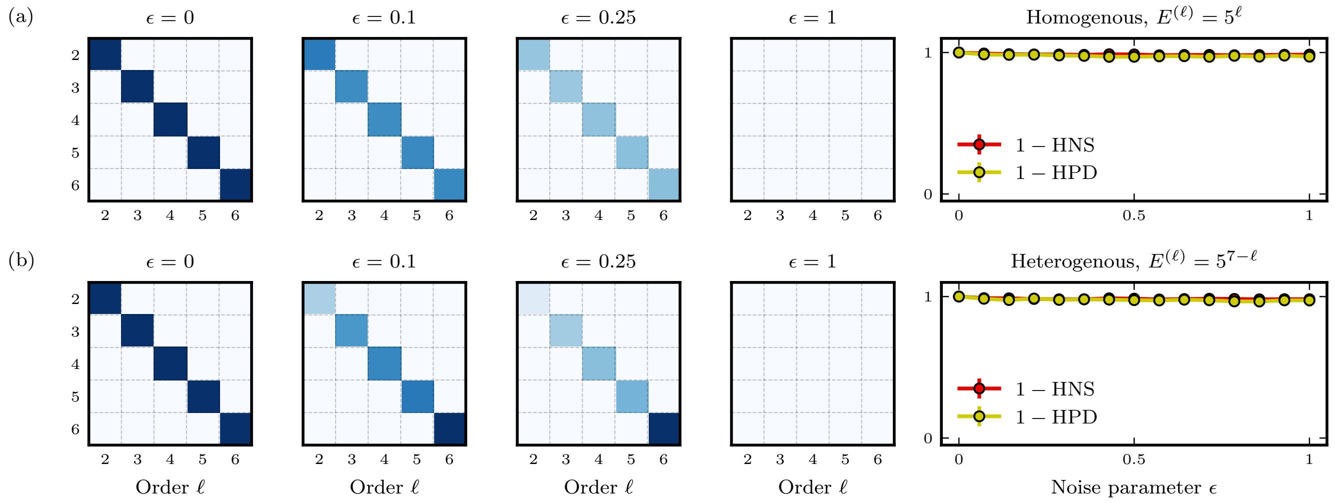

FIG. S12. **Synthetic experimental results for the Hyper NetSimile (HNS) and Hyperedge Portrait Divergence (HPD) distance measures, transformed as $1-$ distance to form similarity measures.**

We also repeat the experiments of Fig. 3 of the main text, in which block-nested hypergraphs are sequentially attacked so as to highlight similarity across orders of interaction. For this, we compare our measure of $\mathrm{NMI}_{\mathrm{cross}}$ against the three measures above for different block configurations (see Fig. S13), finding a similar story. The average Jaccard similarity measure performs relatively well throughout the block disruptions, but still assigns more similarity than warranted. Meanwhile, both $1-\mathrm{HNS}$ and $1-\mathrm{HPD}$ are once again insensitive to any dissimilarity induced by the attacks over blocks.

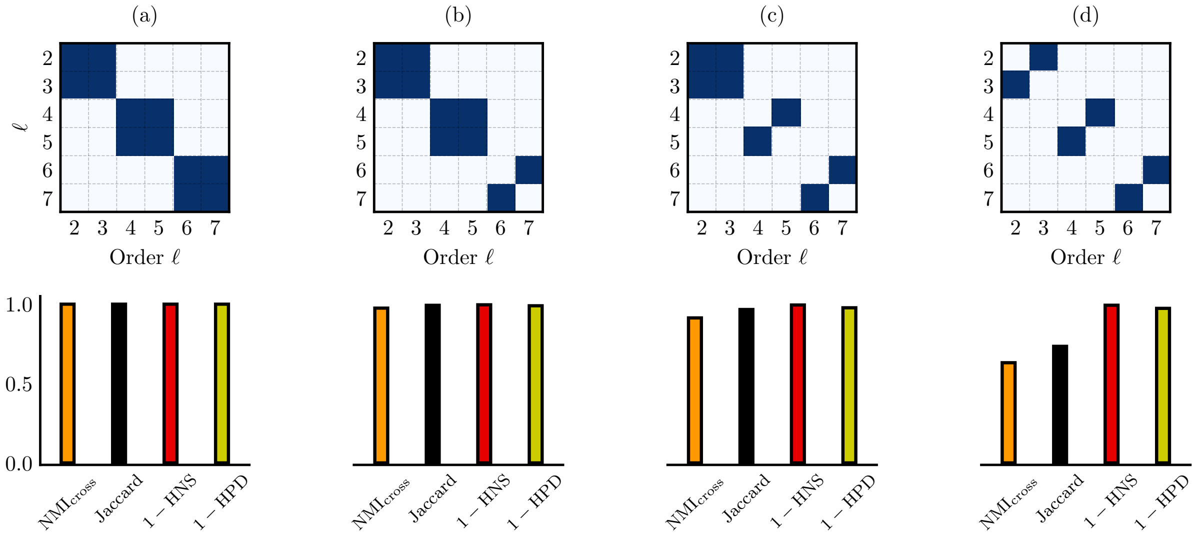

FIG. S13. **Comparison of $\mathrm{NMI}_{\mathrm{cross}}$ with the Jaccard baseline and measures of (28) in the block-randomized hypergraph experiment of Fig. 3 in the main text.**

REFERENCES

1. M. De Domenico, V. Nicosia, A. Arenas, V. Latora, Structural reducibility of multilayer networks. *Nat. Commun.* **6**, 6864 (2015).

2. S. Ok, A graph similarity for deep learning. *Adv. Neural Inf. Process. Syst.* **33**, 1–12 (2020).

3. N. Attar, S. Aliakbary, Classification of complex networks based on similarity of topological network features. *Chaos* **27**, 091102 (2017).

4. P. Wills, F. G. Meyer, Metrics for graph comparison: A practitioner's guide. *PLOS ONE* **15**, e0228728 (2020).

5. N. M. Kriege, F. D. Johansson, C. Morris, A survey on graph kernels. *Appl. Netw. Sci.* **5**, 1–42 (2020).

6. R. Sharan, T. Ideker, Modeling cellular machinery through biological network comparison. *Nat. Biotechnol.* **24**, 427–433 (2006).

7. N. Nikolova, J. Jaworska, Approaches to measure chemical similarity–a review. *QSAR Comb. Sci.* **22**, 1006–1026 (2003).

8. A. Mheich, F. Wendling, M. Hassan, Brain network similarity: Methods and applications. *Netw. Neurosci.* **4**, 507–527 (2020).

9. K. Faust, J. Skvoretz, Comparing networks across space and time, size and species. *Sociol. Methodol.* **32**, 267–299 (2002).

10. R. Lambiotte, M. Rosvall, I. Scholtes, From networks to optimal higher-order models of complex systems. *Nat. Phys.* **15**, 313–320 (2019).

11. F. Battiston, G. Cencetti, I. Iacopini, V. Latora, M. Lucas, A. Patania, J.-G. Young, G. Petri, Networks beyond pairwise interactions: Structure and dynamics. *Phys. Rep.* **874**, 1–92 (2020).

12. F. Battiston, E. Amico, A. Barrat, G. Bianconi, G. Ferraz de Arruda, B. Franceschiello, I. Iacopini, S. Kéfi, V. Latora, Y. Moreno, M. M. Murray, T. P. Peixoto, F. Vaccarino, G. Petri, The physics of higher-order interactions in complex systems. *Nat. Phys.* **17**, 1093–1098 (2021).

13. G. Bianconi, *Higher-Order Networks: An Introduction to Simplicial Complexes* (Cambridge Univ. Press, 2021).

14. C. Bick, E. Gross, H. A. Harrington, M. T. Schaub, What are higher-order networks? *SIAM Rev.* **65**, 686–731 (2023).

15. C. Berge, *Hypergraphs: Combinatorics of Finite Sets* (Elsevier, 1984).

16. I. Iacopini, G. Petri, A. Barrat, V. Latora, Simplicial models of social contagion. *Nat. Commun.* **10**, 2485 (2019).

17. L. Neuhäuser, A. Mellor, R. Lambiotte, Multibody interactions and nonlinear consensus dynamics on networked systems. *Phys. Rev. E.* **101**, 032310 (2020).

18. G. Ferraz de Arruda, A. Aleta, Y. Moreno, Contagion dynamics on higher-order networks. *Nat. Rev. Phys.* **6**, 468–482 (2024).

19. L. Di Gaetano, G. Carugno, F. Battiston, F. Coghi, Dynamical fluctuations of random walks in higher-order networks. *Phys. Rev. Lett.* **133**, 107401 (2024).

20. A. P. Millán, J. J. Torres, G. Bianconi, Explosive higher-order kuramoto dynamics on simplicial complexes. *Phys. Rev. Lett.* **124**, 218301 (2020).

21. Y. Zhang, M. Lucas, F. Battiston, Higher-order interactions shape collective dynamics differently in hypergraphs and simplicial complexes. *Nat. Commun.* **14**, 1605 (2023).

22. A. Civilini, O. Sadekar, F. Battiston, J. Gómez-Gardeñes, V. Latora, Explosive cooperation in social dilemmas on higher-order networks. *Phys. Rev. Lett.* **132**, 167401 (2024).

23. P. S. Chodrow, N. Veldt, A. R. Benson, Generative hypergraph clustering: From blockmodels to modularity. *Sci. Adv.* **7**, eabh1303 (2021).

24. N. Veldt, A. R. Benson, J. Kleinberg, Combinatorial characterizations and impossibilities for higher-order homophily. *Sci. Adv.* **9**, eabq3200 (2023).

25. N. Ruggeri, M. Contisciani, F. Battiston, C. De Bacco, Community detection in large hypergraphs. *Sci. Adv.* **9**, eadg9159 (2023).

26. A. Surana, C. Chen, I. Rajapakse, Hypergraph similarity measures. *IEEE Trans Netw Sci Eng* **10**, 658–674 (2023).

27. A. Martino, A. Rizzi, (Hyper) graph kernels over simplicial complexes. *Entropy* **22**, 1155 (2020).

28. C. Agostinelli, M. Mancastroppa, A. Barrat, Higher-order dissimilarity measures for hypergraph comparison. *J. Complex. Netw.* **14**, cnaf048 (2026).

29. S. Bai, F. Zhang, P. H. Torr, Hypergraph convolution and hypergraph attention. *Pattern Recognit.* **110**, 107637 (2021).

30. R. Feng, T. Xu, X. Xie, Z.-K. Zhang, C. Liu, X.-X. Zhan, A hyper-distance-based method for hypernetwork comparison. *Chaos* **34**, 033140 (2024).

31. S. Saito, Hypergraph modeling via spectral embedding connection: Hypergraph cut, weighted kernel k-means, and heat kernel. *Proc. AAAI Conf. Artif. Intell.* **36**, 8141–8149 (2022).

32. L. Bai, E. R. Hancock, P. Ren, A Jensen-Shannon kernel for hypergraphs. *Lect. Notes Comput. Sci.* **7626**, 181–189 (2012).

33. J. Lugo-Martinez, D. Zeiberg, T. Gaudelet, N. Malod-Dognin, N. Przulj, P. Radivojac, Classification in biological networks with hypergraphlet kernels. *Bioinformatics* **37**, 1000–1007 (2021).

34. D. J. MacKay, *Information Theory, Inference and Learning Algorithms* (Cambridge Univ. Press, 2003).

35. J. Rissanen, Modeling by the shortest data description. *Automatica* **14**, 465–471 (1978).

36. T. P. Peixoto, “Bayesian stochastic blockmodeling,” in *Advances in Network Clustering and Blockmodeling*, P. Doreian, V. Batagelj, A. Ferligoj, Eds. (Wiley, 2019), pp. 289–332.

37. A. Kirkley, Spatial regionalization based on optimal information compression. *Commun. Phys.* **5**, 249 (2022).

38. S. Morel-Balbi, A. Kirkley, Bayesian regionalization of urban mobility networks. *Phys. Rev. Res.* **6**, 033307 (2024).

39. T. P. Peixoto, Reconstructing networks with unknown and heterogeneous errors. *Phys. Rev. X* **8**, 041011 (2018).

40. A. Kirkley, A. Rojas, M. Rosvall, J.-G. Young, Compressing network populations with modal networks reveal structural diversity. *Commun. Phys.* **6**, 148 (2023).

41. R. J. Gallagher, J.-G. Young, B. F. Welles, A clarified typology of core-periphery structure in networks. *Sci. Adv.* **7**, eabc9800 (2021).

42. A. Kirkley, Identifying hubs in directed networks. *Phys. Rev. E.* **109**, 034310 (2024).

43. M. De Domenico, J. Biamonte, Spectral entropies as information-theoretic tools for complex network comparison. *Phys. Rev. X* **6**, 041062 (2016).

44. F. Escolano, E. R. Hancock, M. A. Lozano, M. Curado, The mutual information between graphs. *Pattern Recognit. Lett.* **87**, 12–19 (2017).

45. G. Corso, G. M. Ferreira, T. M. Lewinsohn, Mutual information as a general measure of structure in interaction networks. *Entropy* **22**, 528 (2020).

46. C. Coupette, J. Vreeken, “Graph similarity description: How are these graphs similar?” in *Proceedings of the 27th ACM SIGKDD Conference on Knowledge Discovery & Data Mining* (Association for Computing Machinery, New York, NY, United States, 2021), pp. 185–195.

47. H. Felippe, F. Battiston, A. Kirkley, Network mutual information measures for graph similarity. *Commun. Phys.* **7**, 335 (2024).

48. M. E. Newman, G. T. Cantwell, J.-G. Young, Improved mutual information measure for clustering, classification, and community detection. *Phys. Rev. E.* **101**, 042304 (2020).

49. M. Jerdee, A. Kirkley, M. Newman, Mutual information and the encoding of contingency tables. *Phys. Rev. E.* **110**, 064306 (2024).

50. T. M. Cover, J. A. Thomas, *Elements of Information Theory* (John Wiley & Sons, 2012).

51. F. S. Roberts, B. Tesman, *Applied Combinatorics* (CRC Press, 2024).

52. M. Jerdee, A. Kirkley, M. Newman, Normalized mutual information is a biased measure for classification and community detection. *Nat. Commun.* **16**, 11268 (2025).

53. A. Kirkley, Transfer entropy for finite data. *Phys. Rev. E.* **112**, L052304 (2025).

54. Q. F. Lotito, F. Musciotto, A. Montresor, F. Battiston, Higher-order motif analysis in hypergraphs. *Commun. Phys.* **5**, 79 (2022).

55. T. LaRock, R. Lambiotte, Encapsulation structure and dynamics in hypergraphs. *J. Phys. Complex.* **4**, 045007 (2023).

56. N. W. Landry, J.-G. Young, N. Eikmeier, The simpliciality of higher-order networks. *EPJ Data Sci.* **13**, 17 (2024).

57. L. Gallo, L. Lacasa, V. Latora, F. Battiston, Higher-order correlations reveal complex memory in temporal hypergraphs. *Nat. Commun.* **15**, 4754 (2024).

58. T. LaRock, R. Lambiotte, Exploring the non-uniqueness of node co-occurrence matrices of hypergraphs. arXiv:2506.01479 [cs.SI] (2025).

59. A. Ceria, F. W. Takes, The relevance of higher-order ties. *EPJ Data Sci.* **14**, 62 (2025).

60. N. W. Landry, I. Amburg, M. Shi, S. G. Aksoy, Filtering higher-order datasets. *J. Phys. Complex.* **5**, 015006 (2024).

61. J. Barrett, P. Prałat, A. Smith, F. Théberge, Counting simplicial pairs in hypergraphs. *J. Complex. Netw.* **13**, cnaf021 (2025).

62. A. Kirkley, H. Felippe, F. Battiston, Structural reducibility of hypergraphs. *Phys. Rev. Lett.* **135**, 247401 (2025).

63. M. Lucas, L. Gallo, A. Ghavasieh, F. Battiston, M. De Domenico, Reducibility of higher-order networks from dynamics. *Nat. Commun.* **17**, 1551 (2026).

64. G. Cencetti, F. Battiston, B. Lepri, M. Karsai, Temporal properties of higher-order interactions in social networks. *Sci. Rep.* **11**, 7028 (2021).

65. American Physical Society, APS Data Sets for Research (1.0) [Data set] (2010); https://publish.aps.org/datasets-announcement.

66. Q. F. Lotito, A. Montresor, F. Battiston, Multiplex measures for higher-order networks. *Appl. Netw. Sci.* **9**, 55 (2024).

67. W. Schueller, J. Wachs, V. D. Servedio, S. Thurner, V. Loreto, Evolving collaboration, dependencies, and use in the rust open source software ecosystem. *Sci. Data* **9**, 703 (2022).

68. W. Schueller, J. Wachs, Modeling interconnected social and technical risks in open source software ecosystems. *Collect. Intell.* **3**, 26339137241235614 (2024).

69. L. Betti, L. Gallo, J. Wachs, F. Battiston, The dynamics of leadership and success in software development teams. *Nat. Commun.* **16**, 1–11 (2025).

70. Q. F. Lotito, M. Contisciani, C. De Bacco, L. Di Gaetano, L. Gallo, A. Montresor, F. Musciotto, N. Ruggeri, F. Battiston, Hypergraphx: A library for higher-order network analysis. *J. Complex. Netw.* **11**, cnad019 (2023).

71. Q. F. Lotito, L. Betti, B. Nortier, A. Montresor, F. Battiston, Hypergraphx-data: A repository for higher-order network data. *J. Complex. Netw.* **14**, cnae056 (2026).

72. T. P. Peixoto, A. Kirkley, Implicit models, latent compression, intrinsic biases, and cheap lunches in community detection. *Phys. Rev. E.* **108**, 024309 (2023).

73. A. Kirkley, Inference of dynamic hypergraph representations in temporal interaction data. *Phys. Rev. E.* **109**, 054306 (2024).

74. A. Santoro, F. Battiston, M. Lucas, G. Petri, E. Amico, Higher-order connectomics of human brain function reveals local topological signatures of task decoding, individual identification, and behavior. *Nat. Commun.* **15**, 10244 (2024).

75. M. Neri, A. Brovelli, S. Castro, F. Fraisopi, M. Gatica, R. Herzog, P. A. Mediano, I. Mindlin, G. Petri, D. Bor, F. E. Rosas, A. Tramacere, M. Estarellas, A taxonomy of neuroscientific strategies based on interaction orders. *Eur. J. Neurosci.* **61**, e16676 (2025).

76. N. Ruggeri, F. Battiston, C. De Bacco, Framework to generate hypergraphs with community structure. *Phys. Rev. E.* **109**, 034309 (2024).

77. A. Decelle, F. Krzakala, C. Moore, L. Zdeborová, Inference and phase transitions in the detection of modules in sparse networks. *Phys. Rev. Lett.* **107**, 065701 (2011).

78. I. Iacopini, J. R. Foote, N. H. Fefferman, E. P. Derryberry, M. J. Silk, Not your private tête-à-tête: Leveraging the power of higher-order networks to study animal communication. *Philos. Trans. R. Soc. B Biol. Sci.* **379**, 20230190 (2024).

79. I. Iacopini, M. Karsai, A. Barrat, The temporal dynamics of group interactions in higher-order social networks. *Nat. Commun.* **15**, 7391 (2024).

80. B. Klimt, Y. Yang, "The enron corpus: A new dataset for email classification research," in *European conference on machine learning* (Springer, 2004), pp. 217–226.

81. A. R. Benson, R. Abebe, M. T. Schaub, A. Jadbabaie, J. Kleinberg, Simplicial closure and higher-order link prediction. *Proc. Natl. Acad. Sci. U.S.A.* **115**, E11221–E11230 (2018).

82. J. Diesner, T. L. Frantz, K. M. Carley, Communication networks from the enron email corpus "it's always about the people. enron is no different". *Comput. Math. Organ. Theory* **11**, 201–228 (2005).

83. J. Hardin, G. Sarkis, P. Urc, Network analysis with the enron email corpus. *J. Stat. Educ.* **23**, 1–22 (2015).

84. F. Malizia, S. Lamata-Otín, M. Frasca, V. Latora, J. Gómez-Gardeñes, Hyperedge overlap drives explosive transitions in systems with higher-order interactions. *Nat. Commun.* **16**, 555 (2025).

85. M. Berlingerio, D. Koutra, T. Eliassi-Rad, C. Faloutsos, Netsimile: A scalable approach to size-independent network similarity. arXiv:1209.2684 [cs.SI] (2012).
